# Synergistic Gas-Bubbling and Oxidative Exfoliation for the Reproducible Synthesis of Mesoporous g‑C_3_N_4_ 2D Nanosheets with Enhanced Physicochemical Properties

**DOI:** 10.1021/acsomega.5c13051

**Published:** 2026-02-23

**Authors:** Sajjad Ullah, Livia Eloy da Silva, Elias Paiva Ferreira-Neto, Mohammad Muneeb, Lauro June Queiroz Maia, Yaman Masetto Nicolai, Antônio Claudio Tedesco, Luiz Alberto Beraldo Moraes, Marcos de Oliveira Junior, Beatriz Helena Costa, Rashida Parveen, Sidney J. L. Ribeiro, Rogéria Rocha Gonçalves

**Affiliations:** † Department of Chemistry, Center of Nanotechnology and Tissue Engineering—Mater Lumen Laboratory, Faculty of Philosophy, Science and Letters of Ribeirão Preto, University of São Paulo (FFCLRP-USP), 28133 Universidade de São Paulo, Ribeirao Preto, São Paulo 14040-901, Brazil; ‡ Institute of Chemical Sciences, 600713 University of Peshawar, Faculty of Life and Environmental Sciences, Peshawar 25120, Khyber Pakhtunkhwa, Pakistan; § Institute of Chemistry, 28108 Sâo Paulo State University (UNESP), 14800-060, Araraquara-SP, Brazil; ∥ Grupo Física de Materiais, Instituto de Física-Universidade Federal de Goias, UFG, Goiânia, GO 74605-220, Brazil; ⊥ Department of Chemistry, Center of Nanotechnology and Tissue Engineering-Photobiology and Photomedicine Research Group, Faculty of Philosophy, Sciences and Letters of Ribeirão Preto, University of São Paulo, FFCLRP-USP, Ribeirao Preto, São Paulo 14040-901, Brazil; # Department of Chemistry, Faculty of Philosophy, Science and Letters of Ribeirão Preto, University of São Paulo, FFCLRP-USP, Ribeirao Preto, São Paulo 14040-901, Brazil; ¶ Instituto de Física de São Carlos, Universidade de São Paulo, São Carlos, São Paulo 13566-590, Brazil; ∇ Department of Chemistry, Government Girls Degree College Dabgari, Peshawar, Khyber Pakhtunkhwa 25000, Pakistan

## Abstract

2D nanosheets of graphitic carbon nitride (g-C_3_N_4_) have emerged as promising metal-free photocatalyst. However, their reproducible preparation with a control of physiochemical properties is challenging, and the commonly used thermal polymerization method often leads to the formation of bulk g-C_3_N_4_ with faster e^–^–h^+^ recombination and low surface area, which hinder its full photocatalytic potential. To address these limitations and obtain highly exfoliated and mesoporous 2D nanosheets of g-C_3_N_4_ (EmNs) with desirable physiochemical properties, we propose a facile and reproducible collaborative strategy, based on the synergistic use of ammonium salts as a dynamic gas template and oxidative exfoliation (OE). The prepared EmNs were characterized by an array of complementary analytical techniques including XRD, DRS, EPR, NMR, TEM, SEM-EDX, Raman Spectroscopy, LC-DAD-MS, and time-resolved photoluminescence (PL) measurements and their photoactivity was evaluated through photodegradation of rhodamine B (RhB) dye and 2,4-D herbicide as model pollutants. The proposed one-pot, two-steps thermochemical synthesis protocol not only leads to the preparation of thin (12 ± 3 nm) EmNs but also allows the tuning of their electronic structure, band gap, textural properties, nitrogen vacancies (N-vacancies), and photocatalytic response. Importantly, the band gap energy (*E*_g_), specific surface area, number of N-vacancies, and lifetimes of charge carriers (63–69 ns in EmNs vs 47 ns in pristine g-C_3_N_4_) were all found to increase with increasing NH_4_Cl/melamine ratio and after OE treatment in a synergistic manner. Resultantly, the prepared g-C_3_N_4_ EmNs exhibited 4 times higher photoactivity (*k*_obs._ = 0.09 min^–1^) than pristine g-C_3_N_4_ (*k*_obs._ = 0.023 min^–1^). This one-pot, two-step collaborative strategy can be used as an optimized protocol for the reproducible preparation of thin, highly photoactive, and mesoporous g-C_3_N_4_ nanosheets with tailored and enhanced physicochemical properties for desired applications.

## Graphical abstract

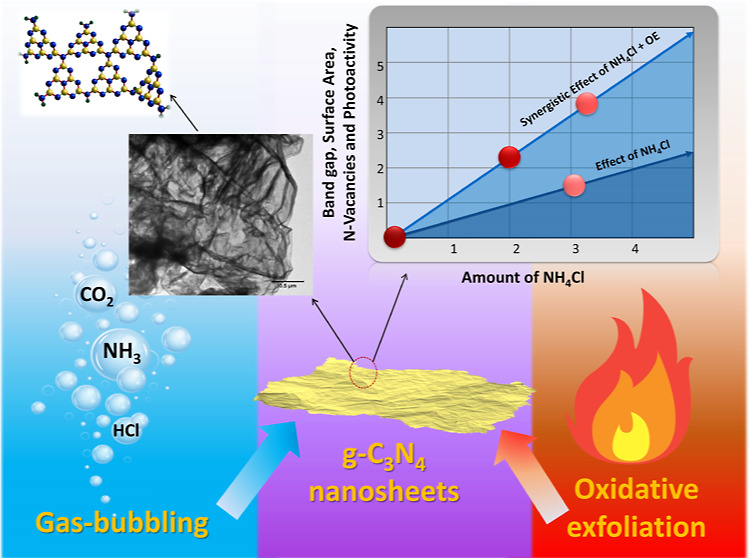

## 1 Introduction

Heterogeneous photocatalysis based on semiconductor photocatalysts is a sustainable approach for addressing the global challenges of environmental pollution^1^ and energy generation.^2^ Some of the attractive and desired features of heterogeneous photocatalysis include high photodegradation efficiency, low cost, availability of various reported synthetic routes for the synthesis of semiconductor photocatalysts with various morphologies and dimensions, and stability of the photocatalysts for recycling and reuse. Furthermore, because of the complete mineralization of organic compounds, there are no problems associated with sludge disposal or the formation of toxic product compounds. However, practical real-world applications of this technology still face challenges, such as photocatalysts’ efficiency, recovery, and upscale and reactor design. Therefore, many research studies have focused on the development of novel photocatalysts with desired physiochemical characteristics, including effective absorption over a broad range (UV–vis–NIR whole-spectrum absorption),^3,4^ high surface area and adsorption capacity, easy recovery and reuse, resistance to poisoning, and facile, low-cost scalable synthesis.

Solar light is an abundant and sustainable source of energy for photocatalytic applications. However, the commonly used traditional photocatalysts, such as TiO_2_ and ZnO, are wide band gap semiconductors that absorb only a limited (∼5–6%) UV region of the solar spectrum. This is why attention has been paid to the development of visible-light-driven photocatalysts such as MoS_2_,^5^ BiOCl, g-C_3_N_4_,^6^ and BiVO_4_,^7–9^ among others.

Among visible light photocatalysts, graphitic carbon nitride (g-C_3_N_4_) has recently emerged as a promising visible-light-driven, metal-free photocatalyst for photocatalytic H_2_ production,^6,10,11^ photodegradation of pollutants and bacterial photoinactivation,^12,13^ CO_2_ reduction to fuels,^14–16^ and water oxidation.^17^ The increased attention paid to this photocatalyst stems from its metal-free nature, visible light photoactivity, adequate thermo-chemical stability, the availability of a variety of relatively low-cost precursors (urea, thiourea, dicyandiamide, and melamine), straightforward synthesis methods, and easy modification.^6,18^ Moreover, the optoelectronic properties of g-C_3_N_4_ can be modified via morphological control.^19^

The most common method for the synthesis of g-C_3_N_4_ is thermal polymerization of the precursors at temperature of 400–600 °C. However, this solid-state reaction often leads to the formation of bulk g-C_3_N_4_ with low surface area. Thus, despite the advantages mentioned above, the full photocatalytic potential of g-C_3_N_4_ is limited by its low surface area, low light absorption, poor adsorption capacity, and fast electron–hole recombination. Different strategies to address these limitations include metal/nonmetal doping,^17,20^ coupling with other semiconductors to form heterojunctions,^13,21^ and structural modifications through calcination,^22^ chemical/thermal exfoliation, and protonation, as reviewed in detail by Saman et al.^6^

One of the best strategies to increase the surface area, decrease electron–hole recombination, and enhance the photocatalytic activity of g-C_3_N_4_ is to prepare two-dimensional (2D) g-C_3_N_4_ thin nanosheets.^12,23–25^ The resulting thin nanosheets exhibit unique and distinct properties such as high surface area, high content of active surface sites and N-vacancies, and short transport distance of photogenerated electrons and holes (to reach the photocatalyst’s surface), leading to enhanced photocatalytic activity.^18,26–28^ Preparation of g-C_3_N_4_ nanosheets is often done by converting bulk g-C_3_N_4_ into nanosheets using a top-down approach.^29^ For instance, ultrathin g-C_3_N_4_ nanosheets have been prepared from presynthesized bulk g-C_3_N_4_ via a liquid (aqueous) phase exfoliation route using water as an exfoliating agent.^30^ Wu et al. converted presynthesized bulk g-C_3_N_4_ into nanosheets using NH_4_Cl-based secondary calcination (400 °C), where the resulting samples showed a clear enhancement in H_2_ evolution performance due to the introduction of amino groups.^29^ The thermal decomposition of NH_4_Cl produces NH_3_ and HCl gases, which help in the delamination and depolymerization of g-C_3_N_4_, thus converting bulk materials into nanosheets.^29^ Using a top-down approach, Niu et al. converted presynthesized bulk g-C_3_N_4_ into 2 nm thick nanosheets by calcination in air, and the resulting nanosheets exhibited higher photocatalytic activity than bulk g-C_3_N_4_ toward H_2_ evolution reaction.^22^

In the present study, we employed a novel, one-pot, two-step collaborative strategy for the controlled preparation of highly exfoliated g-C_3_N_4_ nanosheets (EmNs) with a tailored band gap, mesoporous structure, increased surface area, and enhanced photoactivity. Unlike the top-down approaches mentioned above, which are based on conversion of preprepared bulk g-C_3_N_3_ into nanosheets, we used different amounts of NH_4_Cl, as a chemical bubbler or gas-releasing agent, in an in situ manner, during the bottom-up thermal polymerization of a melamine precursor to directly achieve the formation of partially exfoliated nanosheets, followed by an ex situ secondary oxidative exfoliation (OE) treatment under ambient air^22^ to further/fully exfoliate and refine the physiochemical properties of the prepared g-C_3_N_4_ nanosheets, while keeping the metal-free idea intact. The effect of the precursors and synthesis conditions employed during the thermal polymerization of g-C_3_N_4_ on the morphology, optical, photophysical properties, number of N-vacancies, and photocatalytic activity are discussed to establish a clear synthesis conditions–structure–property relationship. We present a detailed account of how the morphology, electronic structure, N-vacancies, band gap, and photoactivity of g-C_3_N_4_ nanomaterials can be controlled by adjusting the experimental conditions through a synergistic use of NH_4_Cl (in situ) and OE treatment (ex situ). To demonstrate the reproducibility and applicability of the collaborative thermo-chemical exfoliation protocol presented herein, we successfully prepared g-C_3_N_3_ nanosheets using NH_4_HCO_3_ as an alternate gaseous template (producing NH_3_, CO_2_, and H_2_O vapors upon thermal decomposition) and the prepared sample exhibited physiochemical properties similar to those obtained using NH_4_Cl. The synergistic thermo-chemical exfoliation protocol presented in this study may guide further studies for the controlled and reproducible synthesis of g-C_3_N_4_ nanosheets with tailored properties for the desired potential applications such as water remediation, solar-driven H_2_ production, and atmospheric water harvesting through multifunctional hygroscopic-photocatalytic hydrogel nanocomposites.^31^

## 2 Experimental Section

### 2.1 Preparation of Pristine g-C_3_N_4_ and g-C_3_N_4_ Nanosheets (EmNs)

Pristine g-C_3_N_4_ sample (M-0), corresponding to bulk g-C_3_N_4_, was prepared by thermal polymerization of melamine (99%, Sigma-Aldrich USA) at 550 °C for 3 h.^32^ Typically, 5 g of melamine was ground into a fine powder by using a mortar and pestle and transferred to a ceramic crucible. The crucible containing melamine was partially covered by placing another crucible, as a lid, in an inverted position (Scheme 1). The partially covered crucible containing melamine was then placed in a furnace and heated at 550 °C for 180 min (heating rate of 5 °C/min) to thermopolymerize melamine into g-C_3_N_4_. In parallel, structurally modified and exfoliated g-C_3_N_4_ nanosheets (EmNs) were obtained by the same procedure but adding *x* grams (*x* = 0, 2.5, 5, 10 g) of NH_4_Cl (99.5%, Dinamica Brazil) as a bubble-forming/dynamic gas-releasing template^12^ during the first step of the synthesis (Table 1 and Scheme 1). The corresponding samples were coded as M-*x*. To further enhance the degree of exfoliation and obtain high quality nanosheets, the resulting product (M-*x*) was placed in an open crucible and subjected to a secondary oxidative exfoliation (OE) at 500 °C for 2 h under ambient air.^22^ The final g-C_3_N_4_ nanosheet samples, EmNs, were denoted as M-*x*-OE. The entire synthetic procedure is shown in Scheme 1 below. To test the applicability of our synthesis protocol with respect to the chemical bubbler, the same procedure was used to prepare the EmNs sample (M-ABC-5) using NH_4_HCO_3_ as an alternate gaseous template/chemical bubbler.

**Scheme 1:**
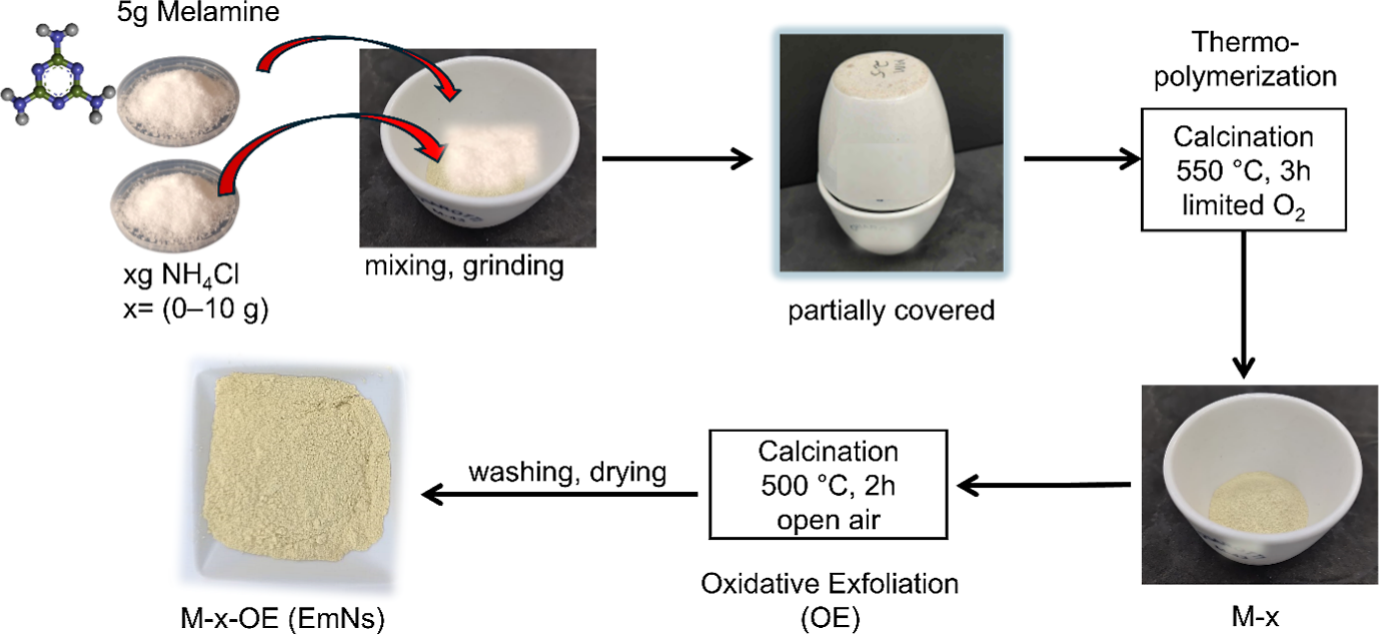
Schematic Representation of the Steps Involved in the Thermopolymerization Synthesis (550 °C, 3 h) of g-C_3_N_4_ Materials (M-*x*) from Melamine (5 g) and NH_4_Cl (*x* g) and after OE (M-*x*-OE) at 500 °C for 2 h under Ambient Air; *x* = Amount of NH_4_Cl = 0–10 g (See Table 1)

**Table 1:** Experimental Conditions Used for the Synthesis of g-C_3_N_4_ Nanosheets (EmNs) by Thermal Polymerization of Melamine at 550 °C for 3 h

sample code	melamine (g)	NH_4_Cl (g)	OE treatment
M-0	5	0	no
M-0-OE	5	0	yes
M-2.5	5	2.5	no
M-2.5-OE	5	2.5	yes
M-5	5	5	no
M-5-OE	5	5	yes
M-10	5	10	no
M-10-OE	5	10	yes
M-ABC-5	5	5 g NH_4_HCO_3_	yes

### 2.2 Characterization Techniques

Solid-state ^13^C NMR was conducted using a Varian Unity INOVA spectrometer operating at 7.05 T (^1^H Larmor frequency of 300 MHz), equipped with a Varian 7 mm MAS (Magic Angle Spinning) probe. The spectra were recorded under MAS conditions with cross-polarization from ^1^H, ^13^C­{^1^H} CP-MAS, using 90° pulses of 6 μs length for ^1^H excitation, recycle delay of 5 s, spinning speed of 5 kHz, contact time of 1 ms, and continuous-wave decoupling during acquisition. ^13^C chemical shifts are reported relative to tetramethylsilane (TMS) using α-glycine as a secondary reference, δ_(C=O)_ = 176.5 ppm.^33^ Scanning electron microscopy (SEM) images of the samples deposited as thin layers on single crystal silicon wafers were obtained, without any metallization, using a Prisma E SEM microscope (Thermo Scientific) operated at 20 keV. Semiquantitative EDS spectra of the samples were obtained by selecting three different regions (60 × 60 μm) of the samples. The Raman spectra were obtained using an XploRA PLUS MicroRaman spectrophotometer (HORIBA France), equipped with a 785 nm laser and coupled to a microscope. X-ray diffractograms were obtained on a Siemens-Bruker D5005-AXS diffractometer (model D2 PHASER) using CuKα radiation (λ = 1.5406 Å), and a graphite monochromator, with a scan rate of 0.5° s^–1^ and a 2θ range ranging from 10° to 60°. For lifetime measurement, the PL decay curves were recorded with an ‘EasyLife X’ time-resolved fluorometer (HORIBA Scientific), equipped with a pulsed picosecond LED source (375 nm). A 400 nm cutoff filter (with 90% transmittance above 430 nm) was used to remove the EMR with λ < 400 nm. Electron paramagnetic resonance (EPR) analysis was performed on a JEOL EPR spectrometer (model JES-FA200, Japan) at an X-band frequency of 9449.5 MHz using the following conditions: center magnetic field (337.3 mT), sweep width 10 mT, scan time 4 min, and amplitude 80. Precisely the same amount (7 mg) of each sample was individually placed in the quartz tube to measure its EPR spectra. For a quantitative comparison of the density of N-vacancies, double integration of the EPR signal (333–341 mT) was performed to calculate the area of the signal. Nitrogen gas physisorption experiments at 77 K were conducted on an automatic N_2_ gas adsorption instrument (ASAP2020) to obtain the specific surface area. Prior to analyses, the powdered samples were heated at 200 °C overnight under vacuum. Specific surface area (A_BET_) values were determined from adsorption isotherms using the Brunauer–Emmett–Teller (BET) method,^34^ in the relative pressure (*P*/*P*_0_) range from 0.05 to 0.30. X-ray photoelectron spectroscopy (XPS) measurements were conducted by using a UNI-SPECS UHV spectrometer equipped with a Mg Kα X-ray source. High-resolution N 1s spectra were collected by using a pass energy of 15 eV and an energy step of 0.05 eV. The data were analyzed by using CasaXPS software. Energy calibration was performed by referencing to the C 1s peak of adventitious carbon at 284.8 eV. Deconvolution of the N 1s spectra was carried out using three peak components with identical full width at half-maximum (FWHM), employing a mixed Gaussian–Lorentzian line shape with 30% Lorentzian contribution.

### 2.3 Photocatalytic Study

The photocatalytic activity of the samples was evaluated through the photodegradation of Rhodamine B (RhB) and 2,4-D herbicides as model pollutants. For this purpose, 20 mg of photocatalysts were dispersed in 20 mL of deionized water and sonicated for 5 min to obtain a homogeneous suspension, and this suspension was added to 20 mL of RhB dye solution or 2,4-D solution (both 20 mg. L^–1^). The mixture was transferred to a cylindrical borosilicate tube reactor (internal diameter = 5 cm) and stirred in the dark for 40 min to allow establishment of adsorption–desorption equilibrium. The pollutant–photocatalyst suspension was then exposed to UV–visible light from a xenon lamp (450 W, power density 611 mW·cm^–2^) placed 20 cm away from the sample in the photoreactor. To evaluate the photocatalytic activity under visible light, a 400 nm cutoff filter was used to remove UV light (λ < 400 nm). Aliquots were withdrawn at regular time intervals, centrifuged at 10,000 rpm, and electronic absorption spectra of the supernatant were taken to follow the degradation of RhB (λ_max_ = 554 nm) or 2,4-D (λ_max_ = 230 nm) and evaluate the photocatalytic activity of the samples. The photodegradation of 2,4-D and its degradation pathway were also studied using an LC-MS hyphenated technique. Direct photolysis (DP) of the RhB dye by light in the absence of any photocatalyst was also investigated under the same conditions.

The recyclability tests were performed by exposing the dye-photocatalyst mixture to UV–visible light from a Xe lamp for 40 min. The used photocatalyst was recovered by allowing the photocatalyst suspension to settle down naturally overnight, discarding the supernatant solution part, and carefully recovering the photocatalyst particles after each cycle. The recovered powder was redispersed in 20 mL of deionized water and exposed to UV–visible light for self-cleaning of its surface. Next, 20 mL of fresh dye solution (20 ppm) was added, and the photocatalytic cycle was repeated. To test the stability of the photocatalysts, XRD patterns of the sample before and after 4 repeated cycles were obtained. To determine the active radicals involved in the photodegradation process, the % photodegradation of RhB in the absence and presence of different radical scavengers (0.1 mmol each) including isopropanol (i-ol), benzoquinone, and EDTA as •OH radicals, O_2_^•–^ radical, and h^+^ scavengers, respectively, was studied.

#### 2.3.1 LC-MS Monitoring of 2,4-D Degradation and Its Degradation Products

Photodegradation of 2,4-dichlorophenoxyacetic acid (2,4-D) and its degradation products was monitored using HPLC-DAD-MS. Mass spectrometry (MS) analyses were performed using an Acquity UPLC (Waters) system equipped with a quaternary pump system and an automatic injector coupled to the Xevo TQS mass spectrometer (Waters) with an electrospray ionization (ESI) source and diode array detector. An Ascentis Express C18 (10 cm × 2.1 mm, 2.7 μm) column was used for chromatographic separation, with 0.1% formic acid as mobile phase A and 0.1% acetonitrile/formic acid as mobile phase B. The mass spectrometer operated in full scan, negative ionization mode.

The degradation products were monitored after different photodegradations times. Aliquots of the degradation medium (500 μL) were extracted by solid-phase extraction (SPE) with cartridges containing the C18 phase (Agilent—Bond Elut, 50 mg, 1 mL). The compounds were eluted with methanol, and the resulting solution was transferred to a vial for HPLC-DAD-MS analysis.

## 3 Results and Discussion

To optimize the synthesis of EmNs with improved physiochemical properties, we first systematically studied the effect of NH_4_Cl amount (0, 2.5, 5, 10 g) as a bubble-forming or frothing agent^12^ and the synergistic OE step on the morphology, nano­(micro)­structure, band gap (*E*_g_), and photoactivity of g-C_3_N_4_ prepared by the thermal polymerization of a fixed amount (5 g) of melamine under otherwise similar conditions (Table 1), as discussed below.

### 3.1 Synergic Effect of NH_4_Cl Amount and OE Treatment on Morphology of EmNs

The effect of NH_4_Cl addition during thermal polymerization on the morphology of g-C_3_N_4_ is evident from a comparison of the SEM images of M-0 and M-5 in Figure 1a,b, respectively. The sample M-0 prepared without the addition of NH_4_Cl presents a smooth surface and solid particles with sharp edges, characteristic of bulk materials (Figure 1a). In contrast, M-5 has a rougher and more textured surface due to the role of NH_4_Cl as a gas-releasing template that induces the partial formation of g-C_3_N_4_ into nanosheets materials (Figure 1b).

**Figure 1:**
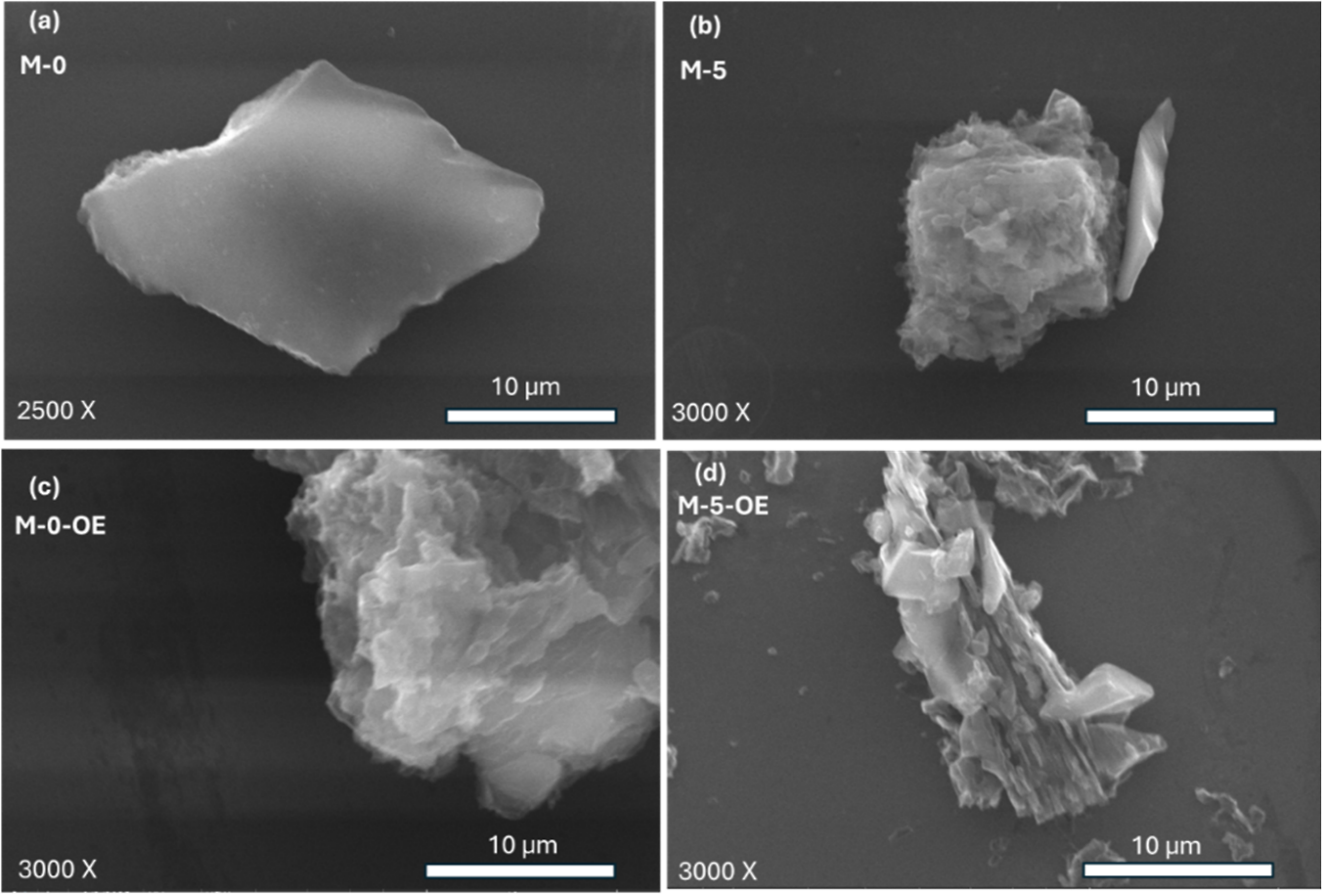
Representative SEM images of the g-C_3_N_4_ samples prepared (a) without NH_4_Cl (M-0) and (b) with NH_4_Cl (M-5), along with images of the corresponding samples (c,d) after OE treatment (lower row). Magnification = 3000× in all images except (a) (2500×).

The g-C_3_N_4_ products obtained after thermal polymerization (550 °C, 3 h) of melamine in the presence of different amounts of NH_4_Cl were further subjected to a synergistic secondary thermal oxidative exfoliation (OE) step at 500 °C for 2 h.^22^ The purpose was to further exfoliate and refine the physicochemical characteristics of partially exfoliated M-*x* samples. The SEM images show that the morphology of both M-0 and M-5 samples changed to a more irregular, textured, and flakier one upon the secondary OE step under ambient air, demonstrating further oxidative exfoliation of the samples (Figure 1c,d, M-0-OE and M-5-OE). This is even more evident from the TEM images of M-0 and M-5 before and after the OE treatment, as shown in Figure 2.

**Figure 2:**
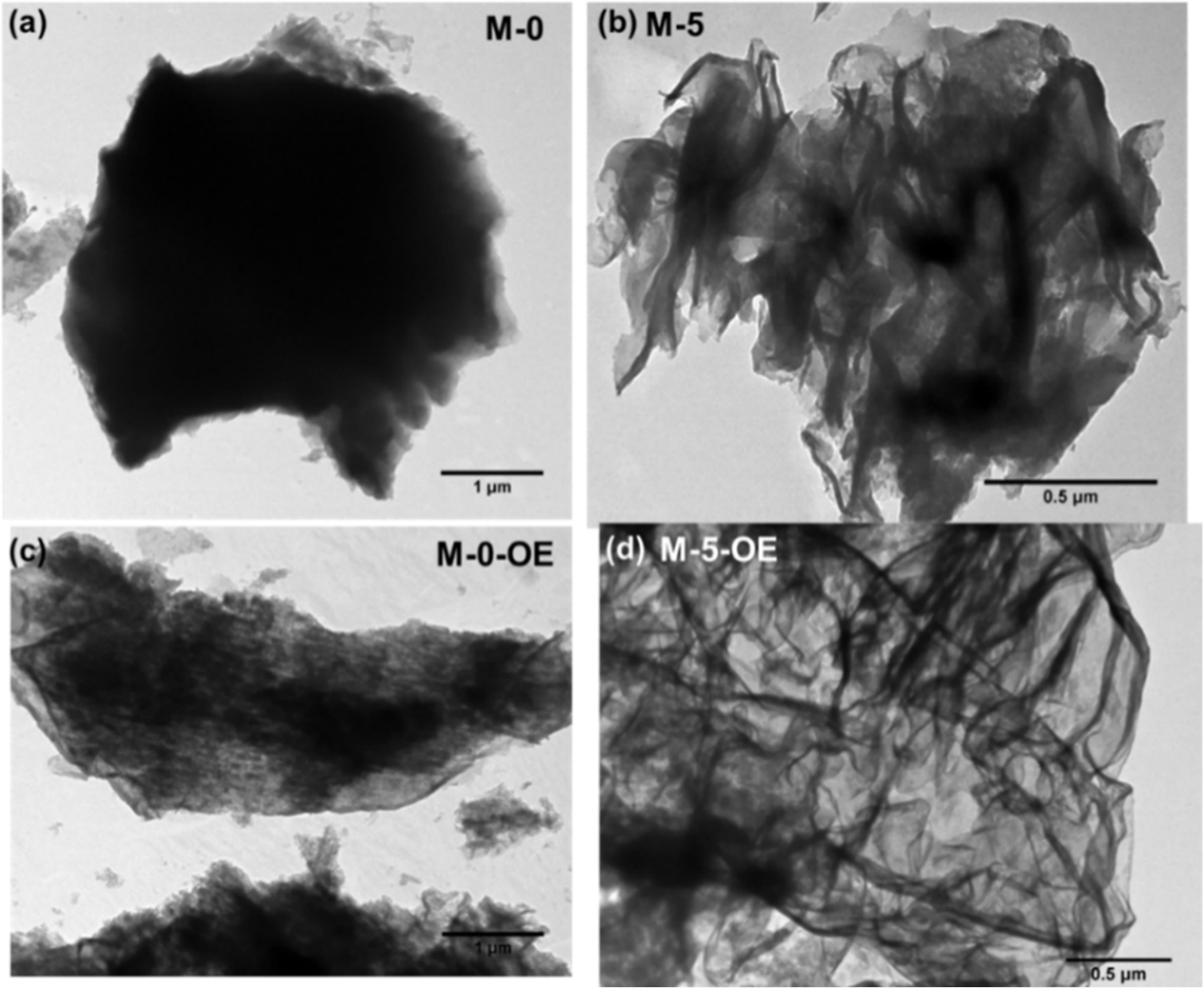
Representative TEM images of g-C_3_N_4_ samples prepared (a) without NH_4_Cl (M-0) and (b) with NH_4_Cl (M-5), along with the corresponding images of these samples (c,d) after OE treatment (lower row).

Further and conclusive evidence of the role of NH_4_Cl as a dynamic gaseous template (to induce the formation of g-C_3_N_4_ nanosheets during thermal polymerization) and OE treatment (in the postsynthesis exfoliation of g-C_3_N_4_) is provided by the TEM images in Figure 2. A comparison of Figure 2a,b shows that while M-0 sample exhibits a dense microstructure typical of bulk materials (Figure 2a), thin g-C_3_N_3_ nanosheets, appearing almost transparent in the TEM images, are clearly observed in case of M-5 (Figure 2b) and M-10 (Figure S1, Supporting Information), confirming the positive role of NH_4_Cl_4_, as a chemical bubbler/gas releasing agent, in the formation of nanosheet morphology.

The influence of NH_4_Cl-induced structural modification and/or exfoliation is also demonstrated by the XRD data (Figure S2). All samples exhibit the characteristic (100) reflection at ∼13° and the (002) reflection at ∼27°,^13,35^ corresponding to the in-plane structural packing and interlayer stacking of the tri-*s*-triazine units, respectively, confirming the formation of nanocrystalline g-C_3_N_4_. However, the samples prepared with the addition of NH_4_Cl show broader and less intense diffraction peaks, indicating a reduction in crystallite size and long-range order due to partial exfoliation, which is consistent with the gas templating effects of NH_4_Cl observed in previous reports.^12,35^ XRD data also confirmed a slightly further broadening of the (002) peak after OE treatment for sample M-5-OE compared to sample M5 (Figure S2). In addition, the Raman mode of bulk g-C_3_N_4_ at 706 cm^–1^ shifts to a slightly lower wavenumber (702 cm^–1^) in nanosheet sample (Figure S3), as previously reported.^36^ This also indicates the formation of thinner nanosheets upon OE treatment, in accordance with the TEM and DRS data. In fact, the average nanosheet thickness of M-5-OE, estimated from the TEM data, was found to be 12 ± 3 nm, thus confirming the nanometric nature of the sample. Furthermore, the nanosheets are apparently thinner in M-5-OE sample prepared using both NH_4_Cl and OE treatment (Figure 2d), advocating the advantage of our collaborative, synergistic synthesis protocol for obtaining g-C_3_N_4_ nanosheets with desired physiochemical properties such as low thickness, higher *E*_g_, and increased photoactivity (vide infra).

### 3.2 Synergistic Effect of NH_4_Cl and OE on Optical Properties of EmNs

The partial NH_4_Cl-induced formation of g-C_3_N_4_ nanosheets during synthesis and/or postsynthesis conversion of the partially exfoliated g-C_3_N_4_ into highly exfoliated nanosheets via synergic OE treatment is reflected in the band gap (*E*_g_) values, measured from Tauc’s plot using diffuse reflectance spectroscopy (Figure 3).

**Figure 3:**
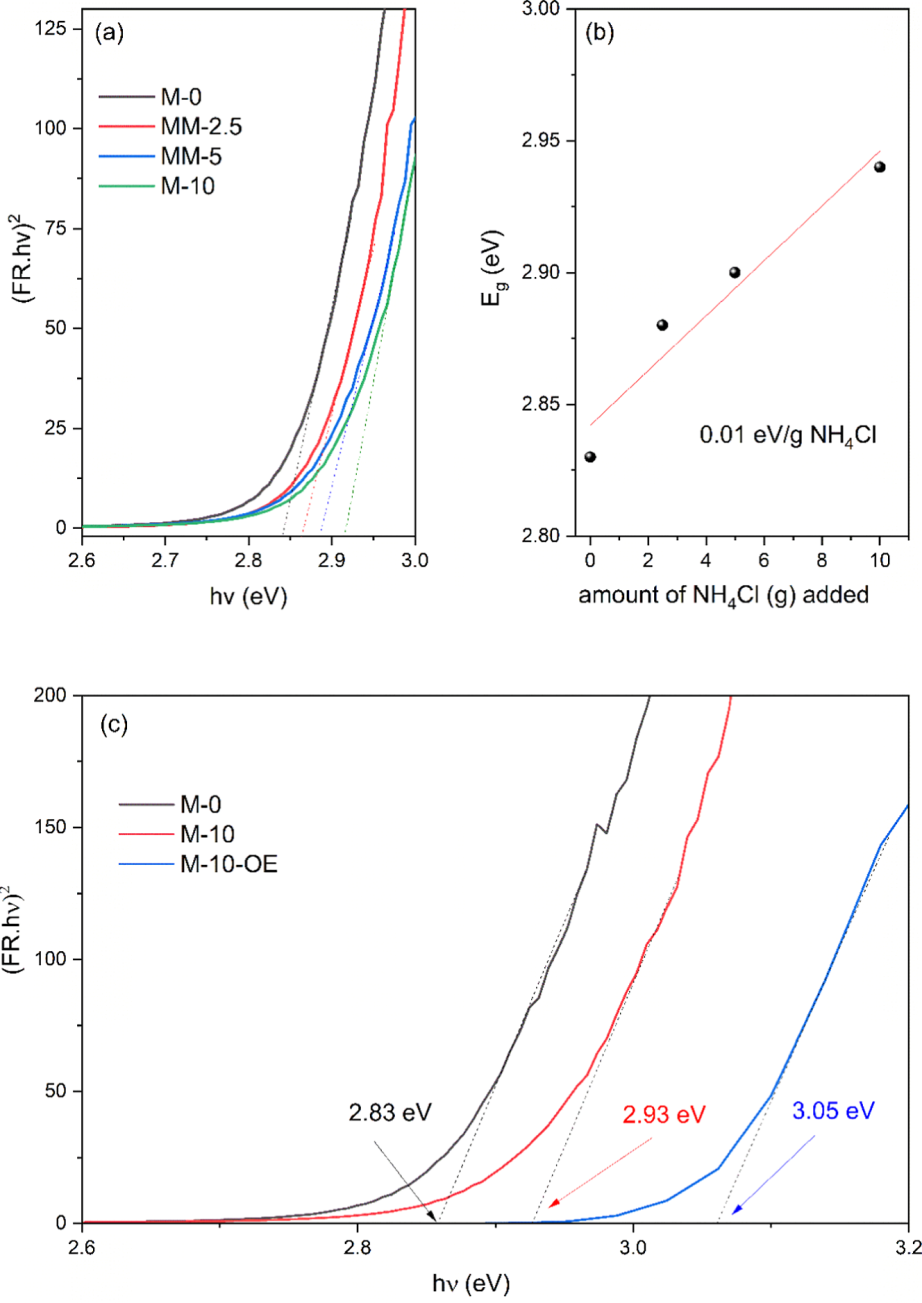
Top panel: Effect of NH_4_Cl addition on the optical properties of g-C_3_N_4_ prepared using a fixed amount of melamine (5 g), varying the amount of NH_4_Cl (0, 2.5, 5, 10 g); (a) Tauc plot and (b) variation of *E*_g_ as a function of the amount of NH_4_Cl. Bottom panel: (c) Tauc plot showing the synergistic effect of both NH_4_Cl and OE on the band gap energy (*E*_g_) of g-C_3_N_4_ sample (M-10) prepared with a higher amount of NH_4_Cl as compared to M-0.

Figure 3a shows that the addition of an increasing amount of NH_4_Cl leads to the formation of nanosheet morphology with progressively wider band gaps. For example, the M-10 sample exhibits a higher band gap of 2.93 eV as compared to the sample prepared without NH_4_Cl (*E*_g_ = 2.83 eV, M-0). In fact, the *E*_g_ values increase almost linearly with increase in the NH_4_Cl amount (Figure 3b), indicating an increasing degree of exfoliation and formation of nanosheets,^19^ as corroborated by TEM, XRD, and Raman spectroscopy data (Figures 2 and S2 and S3). For the M-10 sample, the band gap synergistically increases further to 3.05 eV upon OE treatment with an overall 0.22 eV increase compared to the pristine g-C_3_N_4_ (M-0) sample (Figure 3c). Similarly, for both M-0 and M-5 samples, the structural change to a thin nanosheet morphology and/or increased exfoliation upon OE is accompanied by a further, though subtle, increase in *E*_g_, as shown in Figure S4. Combining experimental and ab initio approaches, Zuluaga et al. observed a similar linear relationship between the structural features of g-C_3_N_4_ and its band gap.^19^ This relationship results from a changing overlap of wave functions associated with the lattice constants, as the structure changes.^19^ Here, we observed the same structure–band gap relationship experimentally, showing that the band gap can be tuned by simply using NH_4_Cl as a gas-releasing/bubbling agent during thermal polymerization of melamine and more so after synergic OE treatment. Such band gap tuning is particularly important for photocatalytic H_2_ production from water.

Table 2 summarizes the *E*_g_ values of samples prepared with and without NH_4_Cl as well as before and after the synergistic OE step. It can be observed from Table 2 and Figure 3c that an overall 0.22 eV increase in the band gap occurs after addition of both NH_4_Cl and the secondary OE step (M-10-OE) as compared to pristine g-C_3_N_4_ (M-0).

**Table 2:** Comparison of the Average Lifetime (τ_av._) of Charge Carriers, Specific Surface Area (*A*_BET_), Band Gap Energy (*E*_g_), and Observed Photodegradation Rate Constants (*k*_obs._) Values for Different g-C_3_N_4_ Samples as a Function of the NH_4_Cl Amount and before and after OE Treatment

sample code	average lifetime (τ_av._)^a^ (ns)	*A*_BET_ (m^2^·g^–1^)	*E*_g_ (eV)	*k*_obs_. (min^–1^)
M-0	47	2.6	2.83	0.023 ± 0.007
M-0-OE	49	22.3	2.85	0.071 ± 0.005
M-2.5	63	–	2.88	0.031 ± 0.005
M-2.5-OE	51.6	–	2.93	0.042 ± 0.003
M-5	69	21.3	2.90	0.054 ± 0.004
M-5-OE	60	58.7	2.94	0.09 ± 0.02
M-10	64	23.8	2.93	0.058 ± 0.006
M-10-OE	61	53.3	3.05	0.083 ± 0.01
M-ABC-5	55	11.1	2.88	0.043

a Average lifetime (ns) of the charge carriers measured from time-resolved recombination fluorescence decay curves.

### 3.3 Effect of NH_4_Cl Addition and Synergistic OE on Photocatalytic Activity

Finally, the effect of structural changes induced by the synergic NH_4_Cl addition–OE treatment on the photoactivity of g-C_3_N_4_ materials was evaluated through photodegradation of RhB and the 2,4-D herbicide. First, direct photolysis (DP) of the dye by the light from the Xe lamp was studied. Figure S5 shows that in the absence of any photocatalyst, a small decrease in the absorption spectra of RhB occurs upon UV–visible illumination and only 12% of the dye was degraded through direct photolysis after 80 min of illumination (Figures S5 and 4a). On the other hand, a drastic decrease in absorbance of the dye and hypsochromic shift of absorption maximum (λ_max_) are observed in the presence of g-C_3_N_4_ nanosheet sample (Figure S6), indicating faster photocatalytic degradation of the dye. The kinetic profiles and observed pseudo-first-order rate constants (*k*_obs._) for pristine g-C_3_N_4_ (M-0) and EmNs samples prepared using varying amounts of NH_4_Cl are compared in Figure 4a,b, respectively. While around 90% of the dye is degraded after 80 min of UV–visible illumination in the presence of the pristine M-0 sample (*k*_obs._ = 0.023 min^–1^), an almost complete (∼100%) degradation of the dye is observed in the case of all g-C_3_N_4_ EmNs samples prepared using NH_4_Cl during the synthesis. The highest and almost equal photoactivity is observed for M-5 and M-10 samples prepared using 1:1 and 1:2 ratios of melamine and NH_4_Cl, respectively. These samples completely degraded the dye in 40–45 min (*k*_obs._ = 0.054–0.058 min^–1^) under UV–visible illumination. Moreover, it took only 18 min for the M-5 sample to photodegrade the same amount of RhB under solar light illumination, registering the highest *k*_obs._ value of 0.164 min^–1^ (Figure S7, blue curve). The same sample exhibited a *k*_obs._ value of 0.021 min^–1^ under visible light (λ > 400 nm) illumination (Figure S7, black curve). Since an increase in the NH_4_Cl amount from 5 to 10 g did not significantly increase the photoactivity, 5 g of NH_4_Cl (or 1:1 ratio of melamine and NH_4_Cl) was found to be the optimum amount, considering cost/benefit ratio. Further increase in photocatalytic activity occurred only after the synergistic OE step as discussed below.

**Figure 4:**
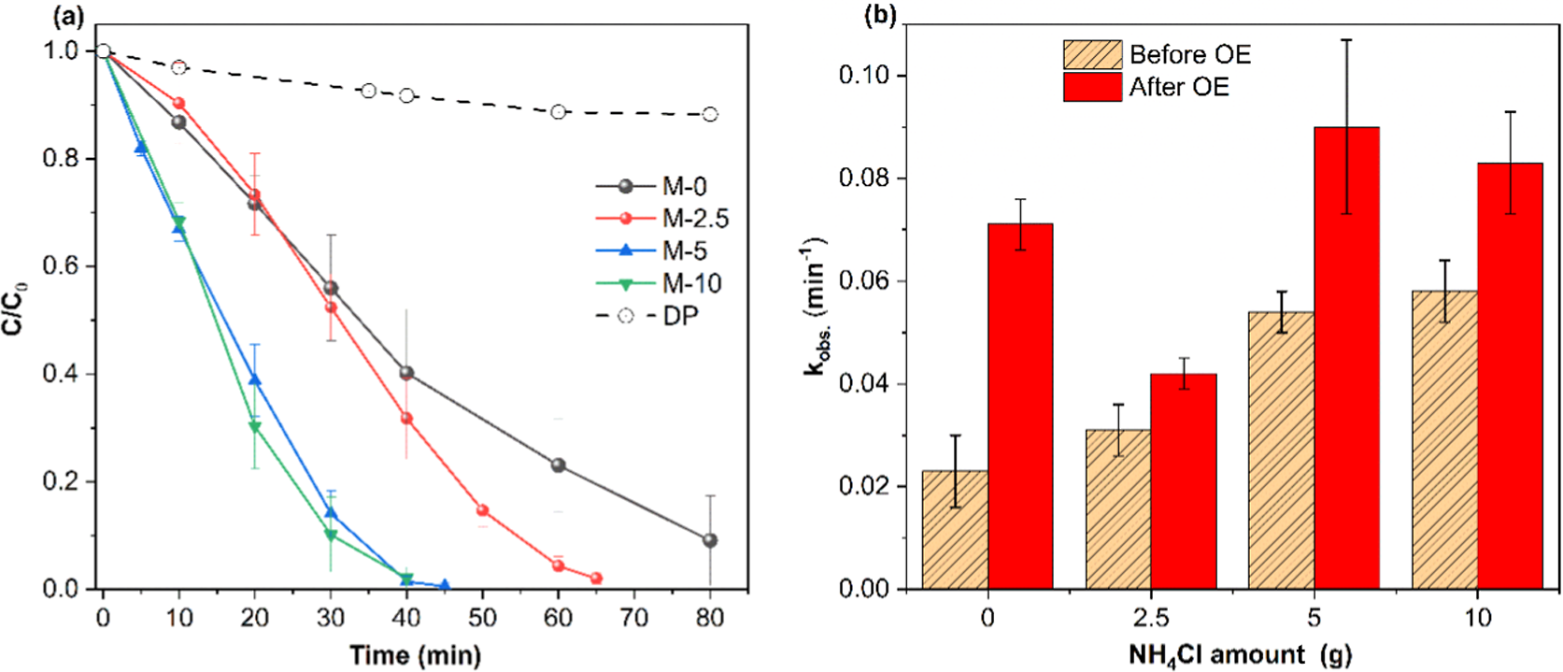
(a) Kinetic profiles and (b) observed rate constants, *k*_obs._, for RhB degradation using g-C_3_N_4_ samples prepared using different amounts (0–10 g) of NH_4_Cl during the thermal polymerization step before (dashed bars) and after (solid bars) OE treatment. Direct photolysis (DP) of RhB by light in the absence of any photocatalyst is also shown in (a). Conditions: RhB = 10 mg. L^–1^, photocatalysts amount = 0.5 g. L^–1^, sample-to-lamp distance = 20 cm.

Next, the effect of the synergetic OE step on the photoactivity of the resulting sample (M-*x*-OE) was studied under identical experimental conditions. A comparison of the photoactivity before and after OE demonstrates that all the samples, including M-0, undergo a synergetic enhancement in photoactivity upon the OE step (Figure 5). The highest, and almost equal, photoactivity was observed for M-5-OE and M-10-OE samples (Figure 5c,d) prepared using high amounts of NH_4_Cl, followed by synergistic OE treatment (Figure 4b, solid bars). These samples completely degraded the dye within 30 min, exhibiting the highest *k*_obs._ value of 0.08–0.09 min^–1^ vs 0.023 min^–1^ of pristine g-C_3_N_4_ (M-0 sample). It can be inferred from Figure 4 and Table 2 that the use of NH_4_Cl alone results in 2.5 times enhancement of photocatalytic activity (*k*_obs._ = 0.023 min^–1^ for M-0 vs 0.058 min^–1^ for M-10) while the synergistic use of NH_4_Cl + OE leads to 3.6 times enhancement in photoactivity for the same sample (*k*_obs._ = 0.023 min^–1^ for M-0 vs 0.083 min^–1^ for M-10-OE). In fact, the effect of NH_4_Cl on *k*_obs._ reaches a saturation point when 5 g of NH_4_Cl is used as indicated by the fact that M-5 (*k*_obs._ = 0.054 min^–1^) and M-10 (*k*_obs._ = 0.058 min^–1^) have similar photoactivity and a significant further increase in *k*_obs._ value occurs only after the OE step (*k*_obs._ = 0.083 min^–1^ for M-10-OE, Figure 5d). In summary, the addition of NH_4_Cl during the preparation of g-C_3_N_4_ is beneficial for obtaining mesoporous and partially exfoliated materials, and even more refined and highly exfoliated materials with higher photoactivity can be obtained after a synergistic OE step. This one-pot, two-step collaborative strategy allows synergistic control of the morphology, band gap, and photocatalytic properties of g-C_3_N_4_ materials, which can be tuned according to the desired applications.

**Figure 5:**
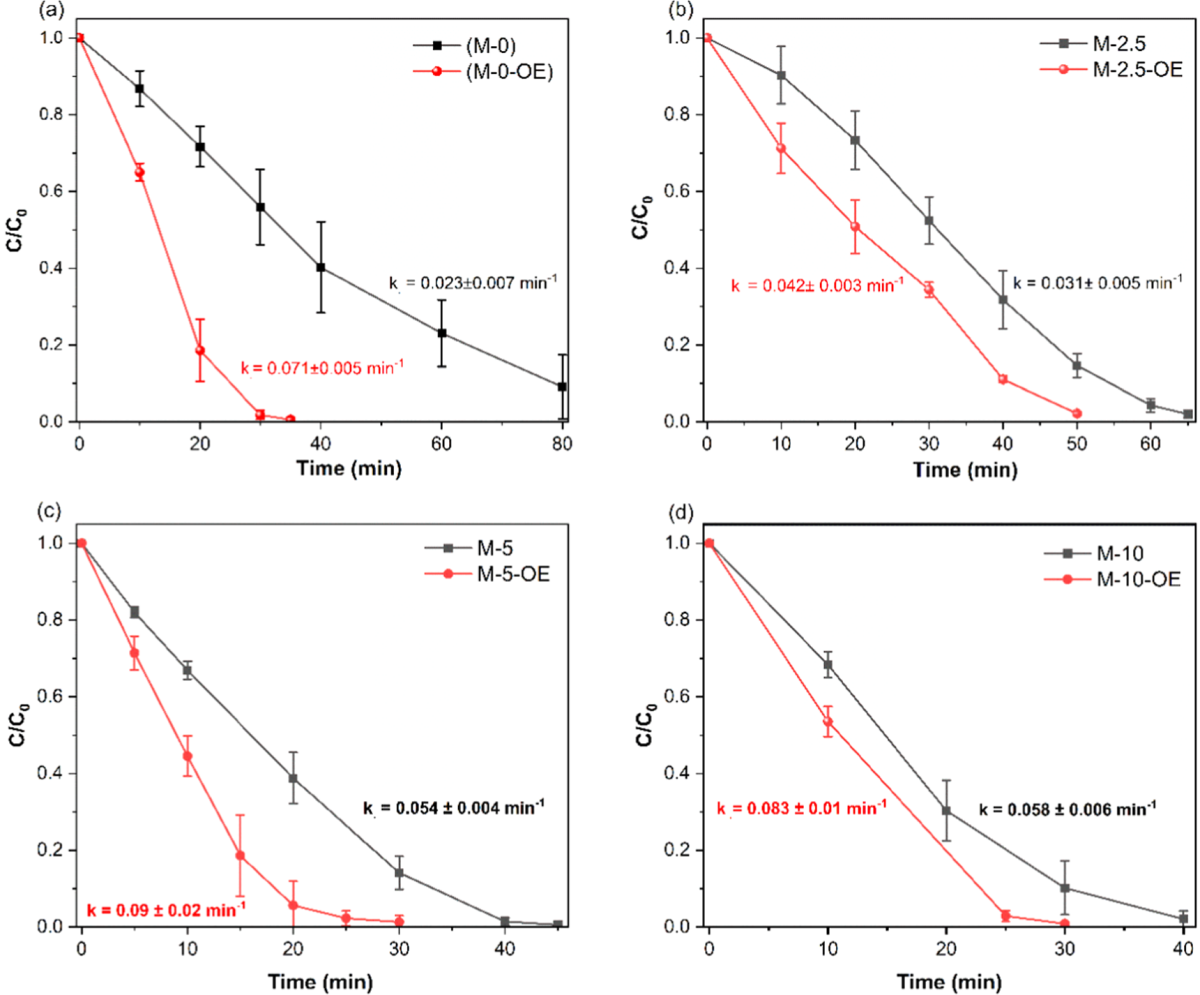
Effect of oxidative exfoliation (OE) on the photocatalytic activity of different g-C_3_N_4_ samples. The photoactivity in terms of kinetic profiles and pseudo-first-order rate constants (*k*_obs._) for each sample before (■) and after (red ●) OE treatment is compared: (a) M-0 vs M-0-OE, (b) M-2.5 vs M-2.5-OE, (c) M5 vs M5-OE, and (d) M-10 vs M-10-OE. Conditions: RhB = 10 mg·L^–1^, photocatalysts amount = 0.5 g·L^–1^, sample-to-lamp distance = 20 cm.

### 3.4 Photodegradation of 2,4-D and Its Degradation Pathways

The photodegradation of persistent herbicide, 2,4-D was also studied in the presence of pristine g-C_3_N_4_ and EmNs sample and followed by LC-MS analysis (Figures S8 and S9) and UV–visible spectroscopy (Figure S10). The ESI­(-)­LC-MS chromatogram before UV–visible illumination shows a single chromatographic peak at a retention time (*T*_R_) of 7 min which corresponds to the 2,4-D molecule (Figure S8). After UV–visible illumination in the presence of the M-5 sample, the intensity of this peak decreases due to degradation of the 2,4-D molecule, and new peaks, corresponding to degradation products, arise at *T*_R_ = 7.3 min (*m*/*z* = 161.96), 6.32 min (*m*/*z* = 177.95), and 5 min (*m*/*z* = 143.99). Importantly, LC-MS analysis shows that 2,4-D (*m*/*z* = 218.8) is almost completely degraded after 60 min of UV–visible illumination, and 03 major subproducts appear (Figure S8). The degradation products were identified from the mass spectra (Figure S9) as follows:

(i) product I (2,4 dichlorophenol (*m*/*z* = 161.96)) formed by the oxidative cleavage of the carboxylate entity of the 2,4-D molecule,
(ii) product II (*m*/*z* = 177.95) formed by further oxidation of 2,4 dichlorophenol,
(iii) product II lost a Cl entity to form 4-chlorocatechol (product III) at *m*/*z* = 143.99.
(iv) other unknown products of smaller masses

Based on LC-MS data, a possible degradation pathway for 2,4-D is presented in Figure 6 below.

**Figure 6:**
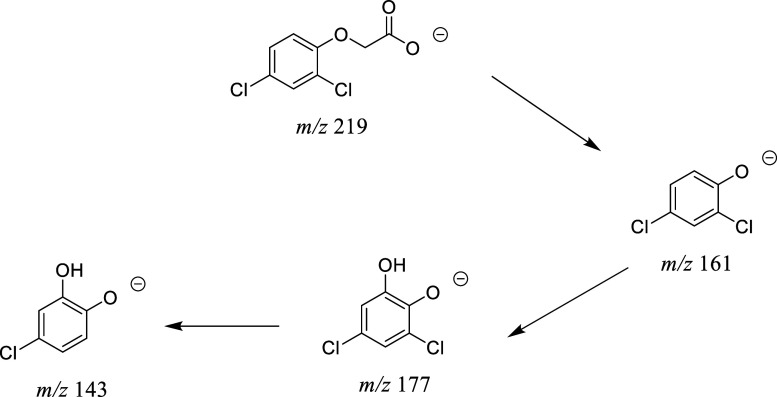
Photocatalytic degradation pathway of 2,4-D molecules.

While LC-MS showed almost complete degradation of 2,4-D in 60 min, the UV–visible spectrophotometry results (Figure S10) show that, after 100 min of UV–visible irradiation, the M-5 and M-0 sample degraded only 77% (*k*_obs._ = 0.035 min^–1^) and 66% (*k*_obs._ = 0.025 min^–1^) of 2,4-D, respectively. The lower degradation of 2,4-D, as measured by UV–visible spectroscopy, may be due to the possible absorption of degradation products at the wavelength of measurements (230 nm for 2,4-D), thus subestimating the % degradation of 2,4-D. It is thus advised to be cautious in the selection of analytical methods for monitoring the photodegradation of target analyte molecules, especially when the absorption of analyte and degradation products may overlap. However, it is important to note from UV–visible spectroscopy data that the M-5 sample prepared using NH_4_Cl demonstrated higher photoactivity than pristine M-0 sample toward the degradation of 2,4-D (Figure S10).

### 3.5 Preparation of g-C_3_N_4_ Nanosheets Using NH_4_HCO_3_ as an Alternate Chemical Bubbler

To test the reproducibility and applicability of the synthesis protocol, we also prepared g-C_3_N_4_ nanosheets using NH_4_HCO_3_ (instead of NH_4_Cl) as an alternate dynamic gas template during thermal polymerization of the melamine precursor. The thermal decomposition of NH_4_HCO_3_ produces gaseous NH_3_, CO_2_, and water vapors which may not only lead to the formation of a porous structure but, together with the effect of thermal oxidation,^22^ also help overcome the cohesive forces (van der Waals forces, hydrogen bonding) between strands of polymeric melon units of the g-C_3_N_4_ sheets and lead to partial depolymerization and the formation of nanosheet morphology. The resulting sample (M-ABC-5) prepared under the same conditions as M-5-OE showed physiochemical characteristics somewhat similar to M-5, except for the *A*_BET_ which was lower (11.1 m^2^·g^–1^) (see Table 2). For instance, like M-5, it showed a clearly porous nanosheet morphology (Figure S11), a bang gap of 2.88 eV (vs 2.90 of M-5) (Figure S12), long lifetime (55 ns) of charge carriers (Figure S13), and an apparent *k*_obs._ (toward RhB photodegradation) of 0.043 min^–1^ (vs 0.054 min^–1^ of M-5) (Figure S14). This suggests that the synthesis protocol is general and reproducible and may be applied to prepare thin g-C_3_N_4_ nanosheets using different chemical bubblers/exfoliating agents (NH_4_Cl, NH_4_HCO_3_, possibly (NH_4_)_2_SO_4_, etc.). The melamine/NH_4_HCO_3_ system is promising and may be studied in detail for the optimized preparation of g-C_3_N_4_ nanosheets.

### 3.6 Why Do Exfoliated Nanosheet Samples (EmNs) Obtained Using NH_4_Cl and Secondary OE Treatment Exhibit Better Photocatalytic Activity?

To answer this question, we first measured the specific surface area (*A*_BET_) from the nitrogen physisorption method^34^ (Figure S15). As can be seen from Table 2, the pristine M-0 sample has a small *A*_BET_ of 2.6 m^2^ g^–1^ while all of the EmNs samples prepared using NH_4_Cl exhibit higher A_BET_ values (21.3–23.8 m^2^ g^–1^). The *A*_BET_ values increase with an increase in the NH_4_Cl amount and after OE treatment due to the exfoliation of g-C_3_N_4_ leading to the formation of EmNs. Importantly, the surface area values increase significantly upon the synergistic secondary OE step under ambient air, demonstrating further oxidative exfoliation of the samples. For instance, the higher *A*_BET_ is found for M-5-OE sample (58.7 m^2^·g^–1^) and M-10-OE (53.3 m^2^·g^–1^) sample due to the synergetic effect of NH_4_Cl and OE. Therefore, it can be concluded that the addition of NH_4_Cl as a chemical bubbler/gas releasing agent followed by the OE step synergistically led to the formation of thin EmNs with 22 times higher specific surface area values and thus around 4 times high photocatalytic activity (Figure 4 and Table 2). Furthermore, the nitrogen adsorption isotherms (type IV) with H4-type hysteresis loops confirm the mesoporous nature of the EmNs sample (Figure S15 and Section S7).^37^ Due to their high surface area and high concentration of exposed active surface sites and defects,^11,38^ the EmNs samples showed higher photocatalytic activity than pristine g-C_3_N_4_. In general, mesoporous materials with a higher surface/volume ratio often exhibit higher active surface area, abundant surface reaction sites, and improved mass transport or diffusion of reactants and products.^39^ Moreover, the mesoporous structure allows better harvesting of light due to multiple internal reflections. Finally, a shorter distance of photogenerated charge carriers in EmNs to reach the photocatalyst’s surface decreases charge carriers’ recombination. These characteristics often contribute to their higher photocatalytic activity than nonporous materials.^39^

Furthermore, to better explain and compare the photoactivity of the samples, we performed time-resolved PL measurements to measure the average lifetimes (τ_av._) of photogenerated charge carriers (e^–^–h^+^ pairs) in different samples prepared without and with different amounts of NH_4_Cl as well as before and after OE treatment. The time-resolved fluorescence decay spectra arising from the radiative recombination of e^–^–h^+^ pairs are compared in Figure 7. In general, all EmNs samples prepared with NH_4_Cl as a dynamic chemical bubbling/exfoliating agent showed slower decay (Figure 7a) and longer charge carriers’ lifetime (Table 2) than pristine g-C_3_N_4_ sample (M-0). The average lifetime (τ_av._) of charge carriers in M-0, M-2.5, M-5, and M-10 was found to be 47 ns, 63 ns, 69 ns, and 64 ns, respectively, testifying to the positive role of NH_4_Cl in preparation of exfoliated g-C_3_N_4_ materials with desired photophysical properties and higher photoactivity.

**Figure 7:**
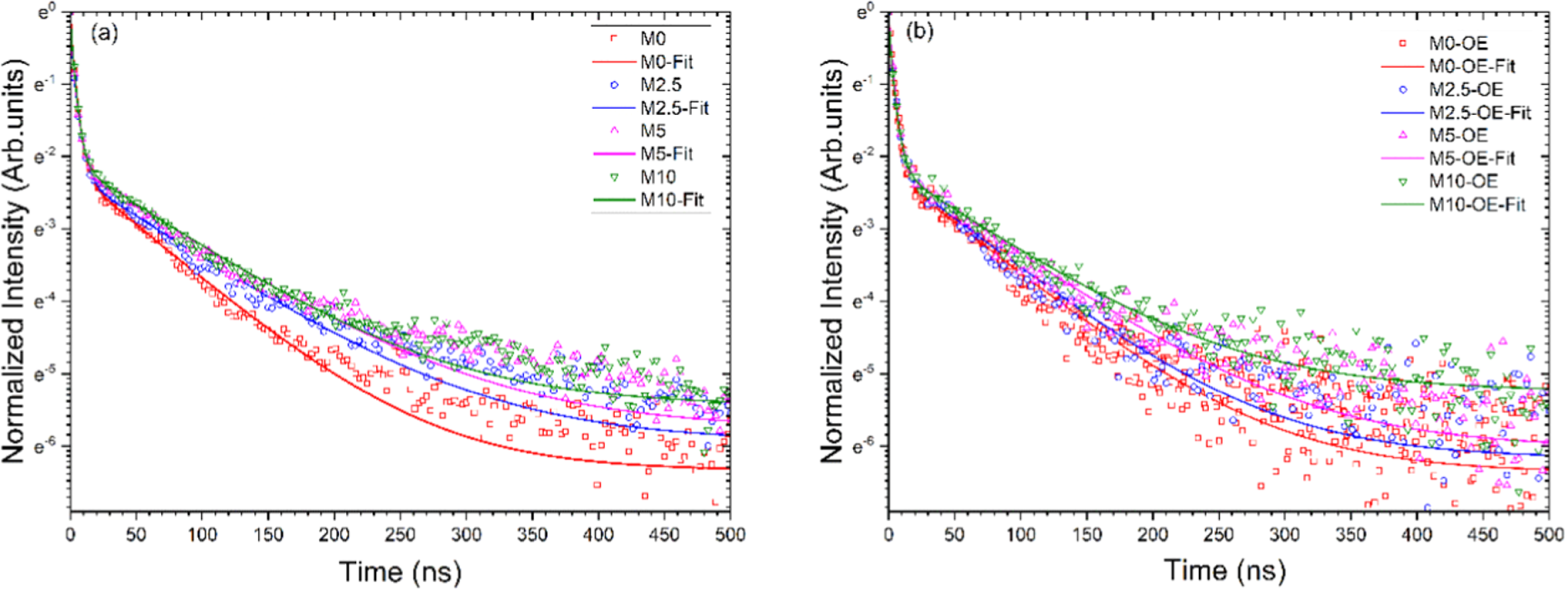
Fitting of the time-resolved recombination fluorescence decay curves for different samples showing the effect of (a) NH_4_Cl content and (b) the OE treatment. The g-C_3_N_4_ samples were photoexcited at a wavelength of 375 nm from a picosecond pulsed LED source.

Table 2 and Figure 7b indicate that the EmNs samples exhibit slightly shorter lifetime values after OE treatment, possibly due to excessive increase in density of N-vacancies (vide infra) that may act as new recombination centers.^11^ In fact, it can be observed from a comparison of Figures S22c and d that the lifetime values decrease slightly with increase in N-vacancies after OE. Based on experimental investigation and DFT calculations, Tu et al. also found that overintroduction of N-vacancies can generate deeper midgap states as the recombination centers and an optimized density of N-vacancies leads to enhanced photocatalytic activity.^11^ However, it is important to note that all EmNs samples, including those undergoing OE treatment, showed longer lifetime values as compared to the pristine M-0 sample (Table 2).

Finally, the recombination fluorescence decay curves for the worst (M-0) and best (M-5) photocatalysts are compared in Figure 8. It is evident that the M-0 sample prepared without any NH_4_Cl exhibits a faster exponential decay than M-5 prepared using a 1:1 wt. ratio of melamine and NH_4_Cl. This clearly indicates slower e^–^–h^+^ recombination in the EmNs sample as compared to pristine g-C_3_N_4_ (M-0). Thinner nanosheet morphology promotes charge carrier separation due to shorter distances these carriers need to travel to reach the photocatalyst’s surface to perform redox reactions. Hence, nanosheets (EmNs samples) exhibit lower bulk recombination and thus higher photoactivity than pristine g-C_3_N_4_ (bulk material).

**Figure 8:**
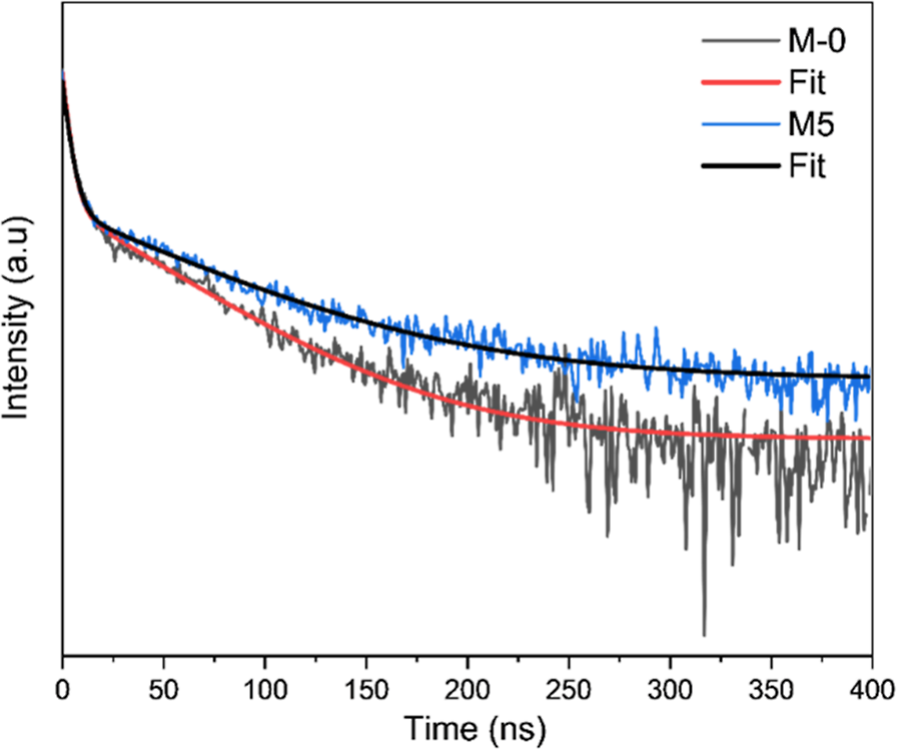
Time-resolved recombination fluorescence decay curves and the corresponding fitting curves for M-0 (black curve, τ_av._ = 47 ns) and M-5 samples (blue curve, τ_av._ = 69 ns).

To further verify this hypothesis and understand the relative photocatalytic activity in terms of charge carriers’ recombination, steady-state photoluminescence (PL) emission intensity of the samples arising from the charge carriers’ recombination was measured (Figure S16). Under 365 nm excitation, PL emissions are observed between 450 and 550 nm with a maximum around 470 nm. In general, all EmNs samples show lower PL intensity than M-0 or M-0-OE (Figure S16a), indicating lower charge carriers’ recombination and hence longer lifetime in EmNs samples, in agreement with the lifetime values obtained using time-resolved PL decay measurement (Figure 7, Table 2). Samples M-5 and M-10 both showed the same reduction (−73%) in intensity compared to sample M-0, which partly explains the similar and better photoactivity of these two samples. However, sample M-5-OE showed a 173% increase in intensity compared to sample M-5 (Figure S16b), possibly due to the formation of N-vacancies (Figure 9, vide infra), which may act as recombination centers. Even so, samples M-5-OE and M-10-OE have an intensity 61% and 76% lower than that of M-0-OE, respectively. That is, reduction in recombination PL emission is observed with addition of NH_4_Cl, irrespective of the OE effect. These results suggest that while NH_4_Cl may increase the photoactivity of EmNs via decreasing the charge carriers’ recombination, OE does the same mainly through surface area increase (Table 2).

**Figure 9:**
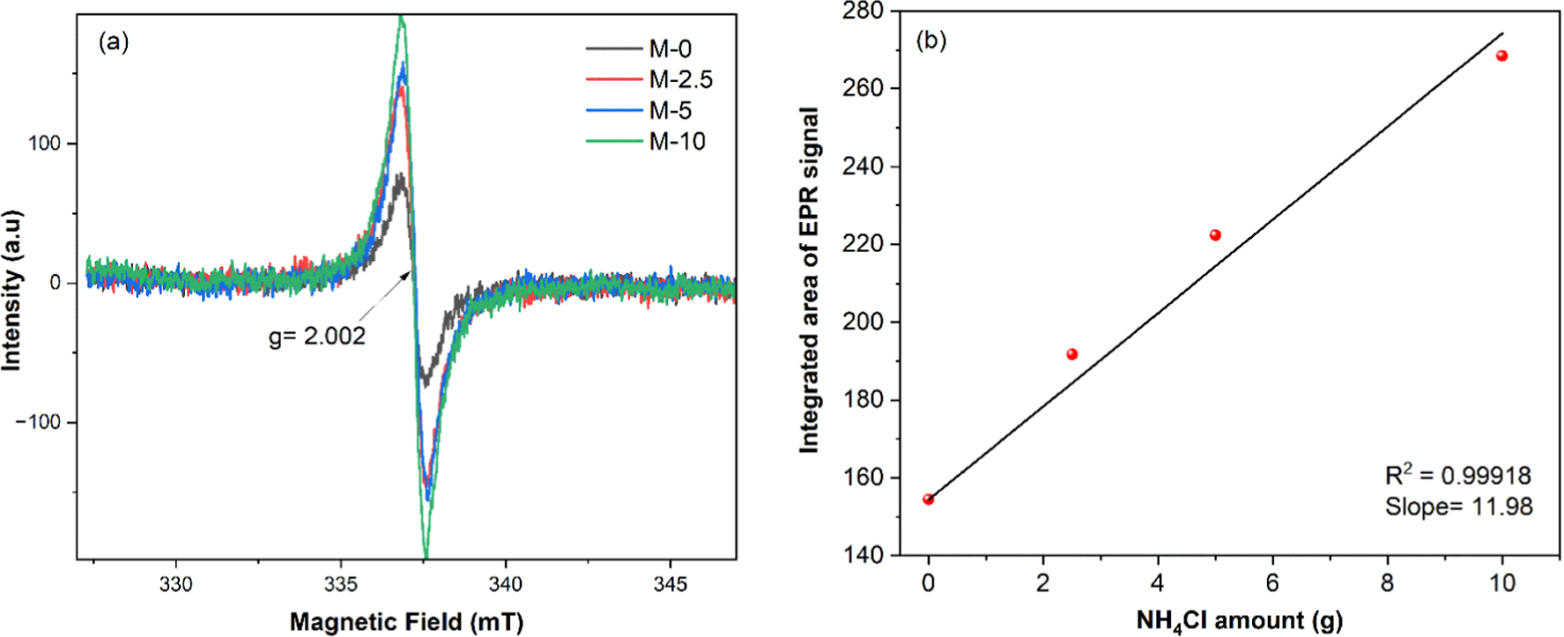
(a) EPR spectra of samples prepared using different amounts of NH_4_Cl during thermal polymerization and (b) integrated area of the EPR signal as a function of NH_4_Cl amount.

Finally, the presence of nitrogen N-vacancies in g-C_3_N_4_ has been found to enhance its photocatalytic performance^11,16,28^ and photocatalytic selectivity.^27^ Considering the importance of vacancies in modulating the photocatalytic response of g-C_3_N_4_, we studied N-vacancies by using EPR analysis (Figure 9). The EPR spectra of all samples show a single Lorentzian line at a *g* value of 2.002 (Figure 9a) which arises from the unpaired electrons on sp^2^ C atoms in the π-conjugated CN aromatic rings. This EPR signal is related to nearby N-vacancies,^11^ and its intensity increases with increase in N-vacancies due to a donation of unpaired electrons from the N-vacancy site to adjacent sp^2^-carbon atoms within π-conjugated aromatic rings in g-C_3_N_4_.^11^ In fact, it can be observed that, as compared to pristine g-C_3_N_4_ (M-0), the intensity of this signal (*g* = 2.002) is higher for all EmNs samples (Figure 9a), indicating a higher density of N-vacancies in the nanosheet samples (EmNs). For a quantitative comparison of the N-vacancies, double integration of the EPR signal was performed to calculate its area and the integrated area was found to linearly increase (*R* ^2^ = 0.99918) with increase in the NH_4_Cl amount (Figure 9b). Moreover, semiquantitative EDS analysis also showed that the N content (atomic %) decreases with increase in the NH_4_Cl amount (Figure S17). These results indicate that NH_4_Cl, as a gaseous template, not only induces the partial formation of nanosheets but also assists in the generation of N-vacancies.

Similarly, the synergic OE treatment is also found to concomitantly and synergistically increase the number of N vacancies further (Figures S18 and S19), which play an important role in the photocatalytic process, as observed earlier (Figures 4b and 5). The N-vacancies, in moderate amount, generate defect states that can trap photogenerated electrons, thereby decreasing the electron–hole recombination,^26^ in agreement with PL data (Figures 7 and 8 and S16). Moreover, these N-vacancies can act as active sites, adsorbing and activating organic pollutants, O_2_ and CO_2_.^16,26^ It has been suggested that N-vacancies can create electron-deficient active surface sites which preferentially interact with electron-rich species (O_2_, aromatic rings, phenolic compounds), thus assisting in their surface redox reactions.^16,26,27^

Furthermore, solid-state NMR spectroscopy was used to further probe the structural modification of the EmNs upon OE treatments. The ^13^C­{^1^H} CP-MAS NMR spectra for investigated samples are depicted in Figure 10, showing two strong resonance signals attributed to the sp^2^-hybridized C_(1)_ carbon atom (C_3N_) and the formation of poly­(tri-*s*-triazine) and C_(2)_ carbon atom (C_2N‑NH*x*_) in the heptazine ring unit associated with sp^2^ carbon atoms linked with terminal –NH*x* groups, respectively.^40–42^

**Figure 10:**
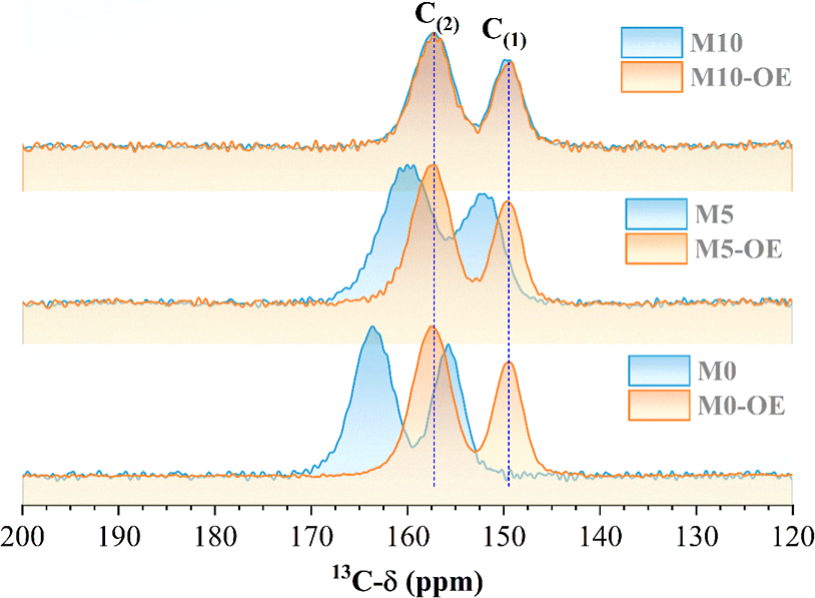
^13^C­{^1^H} CP-MAS spectra of M-0, M-5, and M-10 samples before (blue line) and after (orange line) OE treatment.

For the M-0, M-5, and M-10 samples, the signal C_(1)_ appeared at 155, 151, and 149 ppm and the signal C_(2)_ at 163, 160, and 157 ppm, respectively. On the other hand, for all samples after OE treatment, the signals C_(1)_ and C_(2)_ appeared at 149 and 157 ppm, respectively, and remained unchanged regardless of the NH_4_Cl amount. Therefore, for M-0, M-5, and M-10 samples, a shift of both signals to lower chemical shift values suggests that the addition of NH_4_Cl and the subsequent OE treatment lead to the formation of highly exfoliated g-C_3_N_4_ nanosheets with N-vacancies, which change the electron density of carbon atoms^43,44^ and result in chemical shift variations.

Finally, N 1s XPS measurements were conducted to evaluate the surface chemical states of nitrogen species in the prepared EmNs. The N 1s XPS spectra of the M-0, M-0-OE, M-5, and M5-OE samples are shown in Figure S20. Regardless of NH_4_Cl addition or usage of the OE procedure, all samples display very similar N 1s XPS spectra, which can be satisfactorily fitted using three main contributions. The peak located at 398.4 ± 0.2 eV is assigned to sp^2^-hybridized nitrogen in C–N=C groups in the heptazine rings and the component at 399.7 ± 0.1 eV corresponds to tertiary nitrogen, N–(C)_3_, linking the heptazine units. Finally, the higher binding energy contribution at 400.8 ± 0.1 eV is attributed to C–N–H amino functional groups.^11,29,45,46^ All samples show similar relative concentrations of each nitrogen species (Table S1), indicating that gas bubbling and oxidative exfoliation do not influence nitrogen surface chemical states. This indicates that formation of N-vacancies induced by these treatments does not preferentially affect a specific type of nitrogen site. Such behavior contrasts with the observations reported by Tu et al., which described N-vacancy formation in g-C_3_N_4_ through the preferential removal of sp^2^-hybridized nitrogen species.^11^ On the other hand, our results are consistent with those reported by Yan et al.,^46^ who prepared g-C_3_N_4_ nanosheets via a molten-salt route and observed no significant differences in the N 1s XPS chemical species when compared to bulk g-C_3_N_4._

In summary, the higher photoactivity of EmNs is attributed to its unique physiochemical characteristics including a higher degree of exfoliation, small layer thickness, mesoporous structure, 22 times high surface area, presence of N-vacancies, slightly (0.1–0.22 eV) wider band gap, and longer lifetime (69 ns) of charge carriers or slower electron–hole recombination. Interestingly, many of these physiochemical properties can be tuned by the synergistic use of NH_4_Cl as a chemical bubbler and OE treatment, as demonstrated in this study.

### 3.7 Recyclability and Stability of EmNs

The recyclability of the photocatalysts was studied for 4 consecutive cycles of use (Figure S21 and Section S12). It can be observed from Figure S21a that 98%, 94%, 88%, and 74% photodegradation of RhB occurs within 40 min after the first, second, third, and fourth cycle, respectively. The 24% loss of photoactivity may partly be related to the inevitable loss of photocatalyst powder after each cycle. Moreover, the XRD diffraction patterns of the EmNs sample before and after photocatalytic runs are similar, showing no significant variation or loss of crystalline structure after 4 cycles of use (Figure S21b), indicating good photostability of the material.

### 3.8 Synergistic Effect for Enhancing the Physiochemical Properties of EmNs: A Summary

Figure S22 (Section S13) summarizes the correlation between synthesis conditions, material structure, and physiochemical properties of the prepared g-C_3_N_4_ EmNs and clearly indicates the positive effect of synergistic use of NH_4_Cl and OE treatment on the photodegradation rate constant (*k*_obs._), specific surface area (*A*_BET_), average lifetime (τ_av._) of charge carriers, and density of nitrogen N-vacancies. Notably, most of these properties show an enhancement with an increase in NH_4_Cl amount and after OE treatment. For instance, the *k*_obs._ increases from 0.023 min^–1^ to 0.09 min^–1^ upon addition of NH_4_Cl and OE treatment (Figure S22a). Similarly, the *A*_BET_ for M-0 (2.63 m^2^ g^–1^) increases to 58.7 m^2^ g^–1^ (M5-OE) because of the synergic use of both NH_4_Cl and OE (Figure S22b and Table 2). The same increasing trend is observed in case of N-vacancies (Figure S22d). This aspect of simultaneous control of multiple physicochemical characteristics (*A*_BET_, N-vacancies, *E*_g_, and *k*_obs._) is a prominent and distinct aspect of the present study. As can be seen from Table 2, all the EmNs samples prepared using NH_4_Cl exhibit higher *A*_BET_ values (21.3–23.8 m^2^ g^–1^). The *A*_BET_ values increase with an increase in the NH_4_Cl amount due to partial exfoliation of g-C_3_N_4_ leading to the formation of EmNs. Importantly, the surface area values increase significantly upon the synergistic secondary OE step under ambient air, demonstrating further oxidative exfoliation of the samples. For instance, a higher *A*_BET_ is found for the M-5-OE sample (58.7 m^2^·g^–1^) and M-10-OE (53.3 m^2^·g^–1^) samples due to the synergetic effect of NH_4_Cl and OE.

Although both gas bubble exfoliation and oxidative exfoliation methods have separately been reported before,^12,22^ to the best of our knowledge, a collaborative approach to combine the two for precise and synergistic enhancement of the optoelectronic and many of the physicochemical properties of g-C_3_N_4_ has not been studied and the key novelty of this study is the discovery of synergism when the proposed collaborative strategy is applied. For instance, the use of NH_4_Cl alone results in 2.5 times enhancement in photocatalytic activity (*k*_obs._ = 0.023 min^–1^ for M-0 vs 0.058 min^–1^ for M-10) while the combined use of NH_4_Cl + OE leads to 3.6 times synergistic enhancement in photoactivity for the same sample (*k*_obs._ = 0.083 min^–1^ for M-10-OE). In fact, the effect of NH_4_Cl on *k*_obs._ reaches a saturation point when 5 g of NH_4_Cl is used as indicated by the fact that M-5 (*k*_obs._ = 0.054 min^–1^) and M-10 (*k*_obs._ = 0.058 min^1^) have similar photoactivity, and further significant increase in the *k*_obs._ value occurs only after the OE step (*k*_obs._ = 0.083 min^–1^ for M-10-OE). In fact, a comparison of the photoactivity of our EmNs sample with previously reported g-C_3_N_4_-based photocatalysts shows that our samples are one of the most efficient photocatalysts (Table S2).

The same fact is reflected in the *E*_g_ values of these samples. As compared to M-0 (*E*_g_ = 2.83 eV), M-5 has a 0.07 eV higher band gap and the *E*_g_ value increases only slightly (0.03 eV) in going from M-5 to M-10. Again, a further significant (0.12 eV) increase in the *E*_g_ value occurs only after the OE step (*E*_g_ = 3.05 eV for M-10-OE), advocating the importance of our collaborative approach for tuning the physiochemical properties over an extended range. Moreover, this method ensures reproducible formation of g-C_3_N_4_ nanosheets, which is important from production/applications point of view, especially on a larger scale.

### 3.9 Mechanism of Photodegradation

To determine the photodegradation mechanism and the reactive species involved, we performed radical scavenging assays, as mentioned in the experimental section. While around 100% degradation was observed in the absence of any scavenger, the % degradation decreased in the presence of benzoquinone and EDTA, suggesting that superoxide radicals (*O*_2_^•–^) and holes (h^+^) are mainly responsible for the photocatalytic degradation of RhB (Figure S23), in agreement with previous reports.^47,48^ This makes sense since the N-vacancies can act as active sites, adsorbing and activating molecular O_2_.^16,26^ As mentioned earlier, it has been reported that N-vacancies can create electron-deficient active surface sites which preferentially interact with electron-rich species such as O_2_, thus leading to the formation of superoxide radicals.^16,26,27^ Also, photodegradation of RhB by g-C_3_N_4_ has previously been attributed to the direct hole oxidation.^48^ A proposed mechanism and possible reactions^49^ involved are shown below:

1 $$g‐C_{3}N_{4}+h\upsilon \to {g‐C}_{3}N_{4}^{*}\left(e_{\left(CB\right)}^{-}+h_{\left(VB\right)}^{+}\right),\left(photoexcitation\right)$$

H2O+h(VB)+(g‐C3N4)→OH•+H+⁣(valenceband) 2

$$e_{\left(CB\right)}^{-}\left({g‐C}_{3}N_{4}\right)+O_{2}\to O_{2}^{•-},\left(conduction\;band\right)$$ 3

4 $$O_{2}^{•-}+e^{-}+2H^{+}\to H_{2}O_{2}$$

5 H2O2+e−→O•H+OH−

6 H2O2+O2•−→O•H+OH−+O2

RhB/2,4‐D+h+/O•H→oxidationproducts⁣(photo‐oxidation) 7

The g-C_3_N_4_ is photoexcited by UV–visible light (hυ > *E*_g_), leading to the formation and separation of the electron–hole (e^–^–h^+^) pair and the electron is photoexcited from the valence band (VB) to conduction band (CB) leaving the h^+^ in the VB (eq 1). The h^+^ in the valence band react with water to form ^•^OH radicals (eq 2), while electrons in the CB react with molecular O_2_ to form a superoxide radical anion, O_2_^•^, (eq 3) which is further reduced to H_2_O_2_ (eq 4) and the resulting H_2_O_2_ can be reduced to ^•^OH (eq 5). As a consequence of these reactions (eqs 1–6), reactive oxygen species (^•^OH, O_2_^•–^) and oxidative h^+^ are produced which take part in the photocatalytic degradation of pollutants (eq 7).

## 4 Conclusions

A systematic study of the effect of NH_4_Cl as a gaseous template during (in situ) melamine-based thermo-polymerization of g-C_3_N_4_, followed by a synergistic oxidative exfoliation (OE) of the thermo-polymerized product, was conducted to optimize the synthesis of exfoliated, mesoporous g-C_3_N_4_ nanosheets (EmNs). It was found that addition of NH_4_Cl and subsequent OE treatment synergistically led to the formation of highly exfoliated, mesoporous g-C_3_N_4_ 2D nanosheets (EmNs) with a controlled band gap, a 22 times high surface area (58.7 m^2^·g^–1^), a large number of N-vacancies, and around 4 times higher photocatalytic activity. The band gap, specific surface area, and the density of N-vacancies were found to generally increase with increasing NH_4_Cl amount and after OE, suggesting the formation of thinner g-C_3_N_4_ with an altered electronic structure, as confirmed by microscopic, PL, and DRS analysis. The EmNs samples (M-5-OE and M-10-OE) showed 4 times higher photoactivity than pristine g-C_3_N_4_ (100% photodegradation of RhB dye in 30 min (*k*_obs._ = 0.08–0.09 min^–1^) as compared to 76% degradation in 80 min for the bulk sample M-0 (*k*_obs._ = 0.023 min^–1^)). The one-pot, two-step collaborative method synthesis strategy reported here allows synergistic control of the nanostructure, band gap, surface area, number of N-vacancies, and hence photocatalytic activity and may be used to prepare tailored g-C_3_N_4_ nanomaterials for diverse applications.

## Supplementary Material



## Notes

TEM images of S-10 and M-ABC-5; XRD patterns of EmNs; Raman spectrum of nanosheets vs bulk g-C_3_N_4_; Tauc plots of EmNs; absorption spectrum of RhB showing direct photolysis and photocatalytic degradation; photodegradation of RhB by M-5 EmNs under solar light, UV–visible light, and visible light; ESI (−)­LC-MS chromatograms and mass spectra of 2,4-D; UV–visible spectra of 2,4-D for monitoring its photocatalytic degradation; time-resolved recombination fluorescence decay curves for M-ABC-5 sample; adsorption and desorption nitrogen gas isotherms; steady-state PL emission spectra and XPS, EPR, and EDS measurements of EmNs; recyclability and stability data of EmNs; and active radicals scavenging data (PDF)
