# Supplementary material for: Synergistic Gas-Bubbling and Oxidative Exfoliation for the Reproducible Synthesis of Mesoporous g‑C3N4 2D Nanosheets with Enhanced Physicochemical Properties
Source: ACS Omega. 2026 Feb 23;11(9):15402–16. doi: 10.1021/acsomega.5c13051 (PMC12980174; doi:10.1021/acsomega.5c13051)
Supplement: Supplementary file 1 [file ao5c13051_si_001.pdf]

Supporting Information

Synergistic gas-bubbling and oxidative exfoliation for the reproducible synthesis of mesoporous g-C_3N_4 2D nanosheets with enhanced physicochemical properties

Sajjad Ullah[1,2], Livia Eloy da Silva[1], Elias Paiva Ferreira-Neto[3], Mohammad Muneeb[2], Lauro June Queiroz Maia[4], Yaman Masetto Nicolai[1], Antônio Claudio Tedesco[5], Luiz Alberto Beraldo Moraes[6], Marcos de Oliveira Junior[7], Beatriz Helena Costa[7], Rashida Parveen[8], Sidney J. L. Ribeiro[3], Rogéria Rocha Gonçalves[1*]

[1]Universidade de São Paulo, Department of Chemistry, Center of Nanotechnology and Tissue Engineering - Mater Lumen Laboratory, Faculty of Philosophy, Science and Letters of Ribeirão Preto, University of São Paulo (FFCLRP-USP), Ribeirao Preto, SP, 14040-901, Brazil

[2]Institute of Chemical Sciences, University of Peshawar, Faculty of Life and Environmental Sciences, Peshawar, 25120, KP, Pakistan

[3] Institute of Chemistry, Sâo Paulo State University (UNESP), 14800-060, Araraquara-SP, Brazil

[4] Instituto de Física-Universidade Federal de Goias, UFG, Grupo Física de Materiais, Goiânia, GO, 74605-220, Brazil

[5] Department of Chemistry, Center of Nanotechnology and Tissue Engineering-Photobiology and Photomedicine Research Group, Faculty of Philosophy, Sciences and Letters of Ribeirão Preto, University of São Paulo, FFCLRP-USP, Ribeirao Preto, SP, 14040-901, Brazil.

[6] Department of Chemistry, Faculty of Philosophy, Science and Letters of Ribeirão Preto, University of São Paulo, FFCLRP-USP, Ribeirao Preto, SP, 14040-901, Brazil.

[7] Instituto de Física de São Carlos, Universidade de São Paulo, São Carlos, SP, 13566-590, Brazil

[8] Department of Chemistry, Government Girls Degree College Dabgari, Peshawar, Khyber Pakhtunkhwa 25000, Pakistan

***Corresponding authors:** *Rogéria R. Gonçalves (rrgoncalves@ffclrp.usp.br)*

S1: TEM ANALYSIS OF EmNs

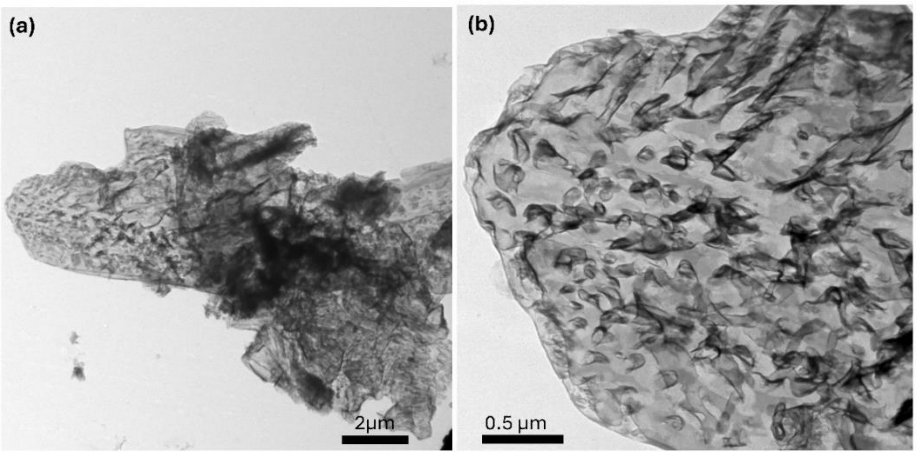

Fig. S1: Representative TEM images of M-10 g-C_3N_4 samples prepared using highest amount (10 g) of NH_4Cl in (a) low magnification (2.5K X) and (b) high magnification (12K X)

S2: XRD ANALYSIS

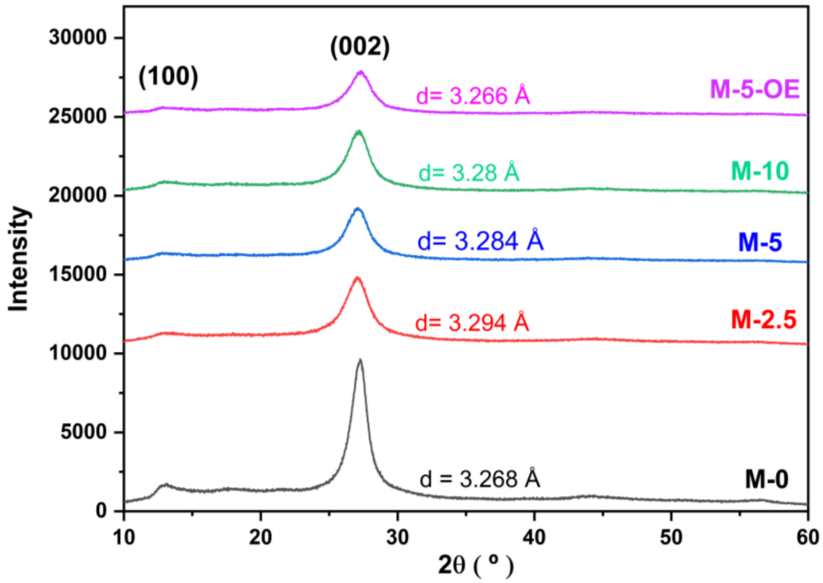

Fig. S2: X-ray diffractograms of EmNs nanosheet samples as compared to pristine g-C_3N_4. The d-values correspond to the (002) planes (2-theta ~27°).

S3: RAMAN SPECTROSCOPY ANALYSIS

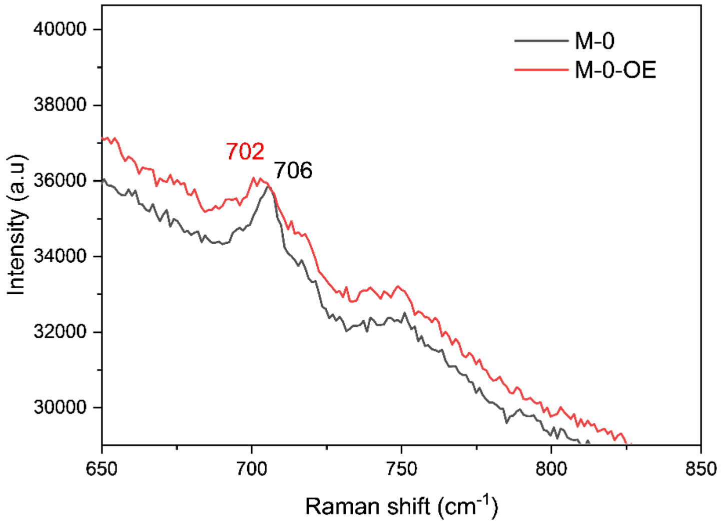

Fig. S3: Raman spectra of bulk M-0 before and after OE treatment. The Raman mode of bulk g-C_3N_4 (black curve) at 706 cm^{-1} shifts to slightly (by 4 cm^{-1}) lower wavenumber (702 cm^{-1}) in nanosheet sample (red curve), as observed in previous report [1].

S4: OPTICAL PROPERTIES

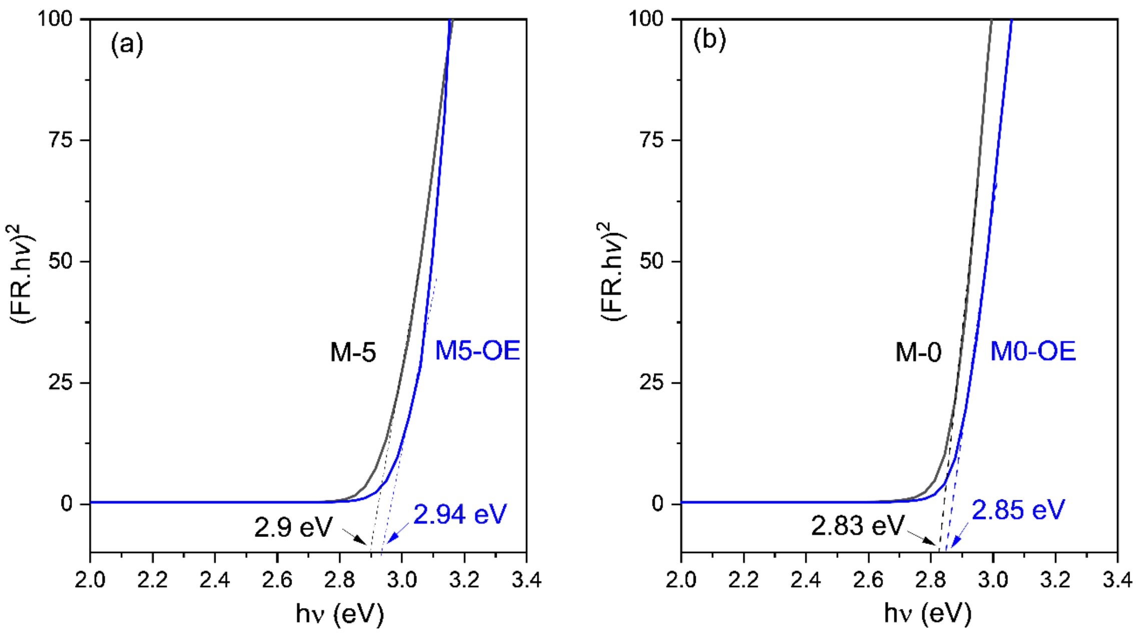

Fig. S4: Tauc's plot, showing the effect of OE treatment on the energy gap (E_g) of g-C_3N_4 samples: (a) (M-5 vs. M-5-OE) and (b) (M-0 vs. M-0-OE)

S5: PHOTOCATALYTIC ASSAYS

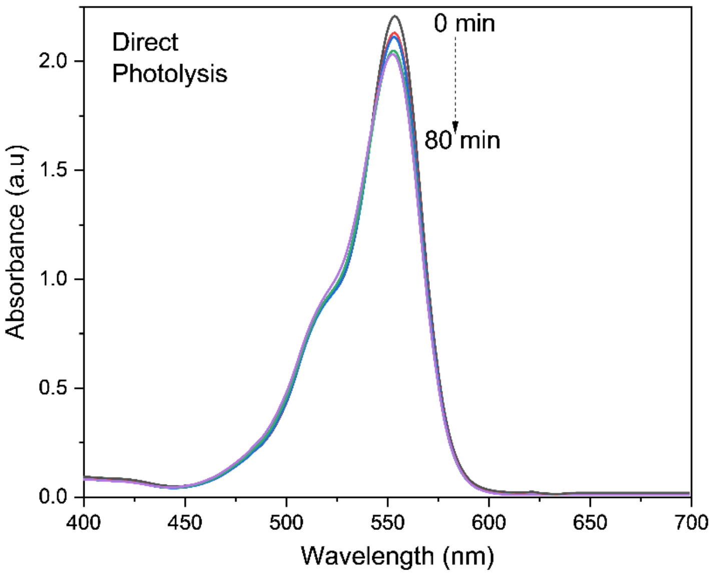

Fig. S5: Electronic absorption spectra of RhB dye (10 mg.L^{-1}) as function of UV-visible illumination time (0-80 min) showing around 12% degradation of the dye in 80 minutes through direct photolysis by light from the Xe lamp in the absence of any photocatalyst.

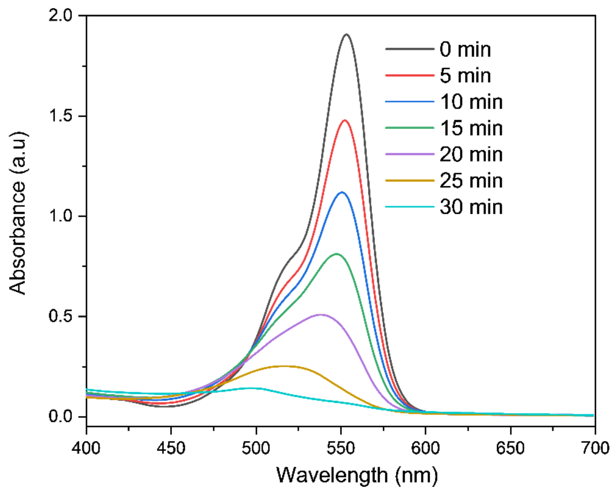

Fig. S6: Electronic absorption spectra of RhB dye (10 mg.L^{-1}) as function of UV-visible illumination time (0-30 min) in the presence of M-5-OE sample. Conditions: RhB = 10 mg.L^{-1}, Photocatalysts amount 0.5 g/L, Xe Lamp (450 W), Sample-to-lamp distance = 20 cm

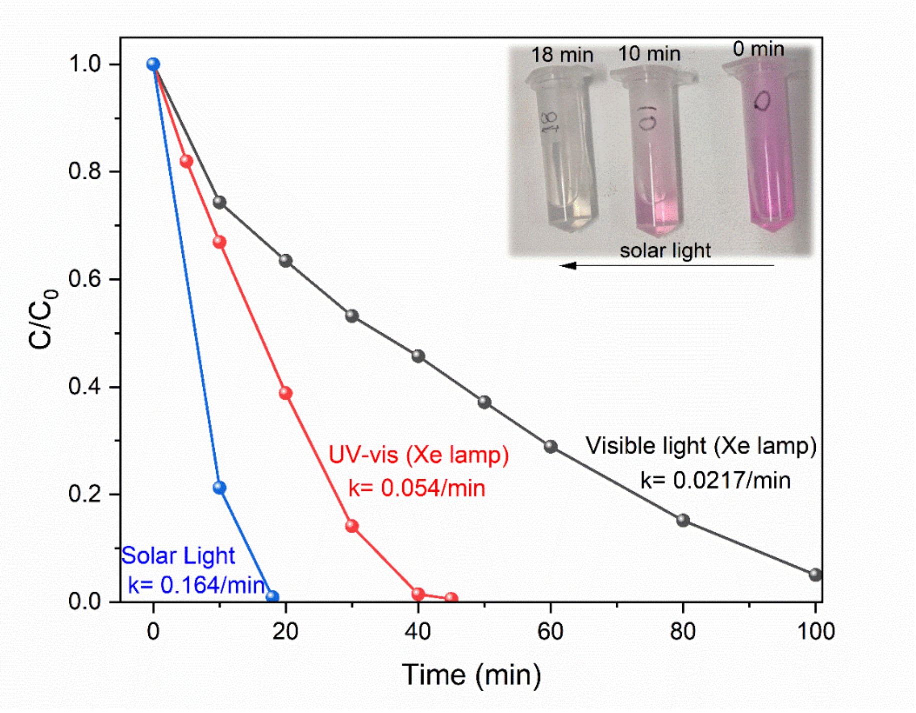

Fig. S7: Photodegradation of RhB by M-5 sample under visible light (λ> 400 nm, black curve) and UV-visible light (red curve) from Xenon lamp and under natural sunlight illumination (blue curve). Inset shows digital photographs of the dye solution after exposure to natural sunlight for different time periods.

S5.1: PHOTODEGRADTION OF 2,4-D:

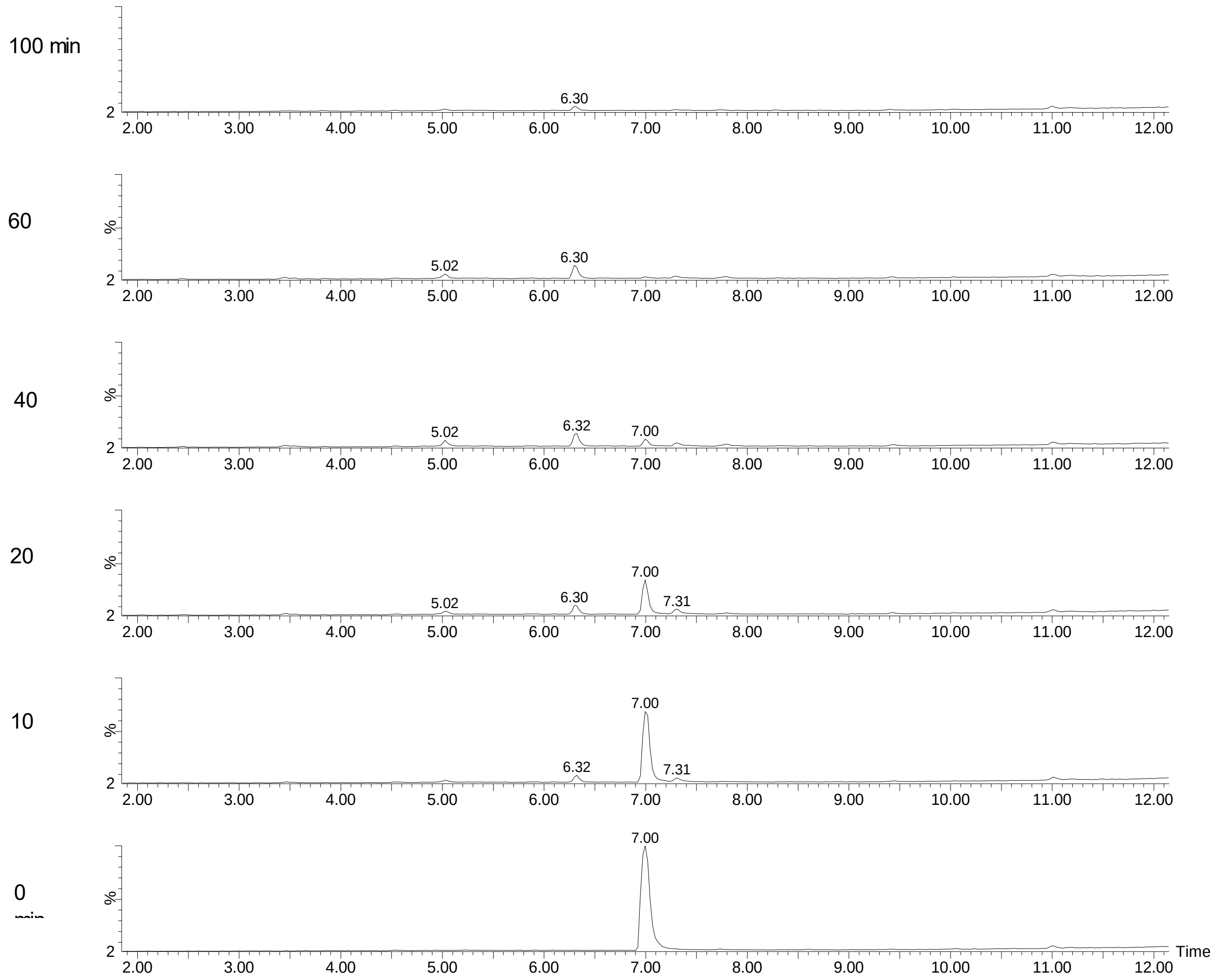

Fig. S8: ESI (-)LC-MS chromatograms of the sample aliquots taken before (bottom, 0 min) and after different time intervals (10, 20, 40, 60, 100 min) of UV-visible illumination in the presence of M-5 EmNs sample showing the photocatalytic degradation of 2,4-D.

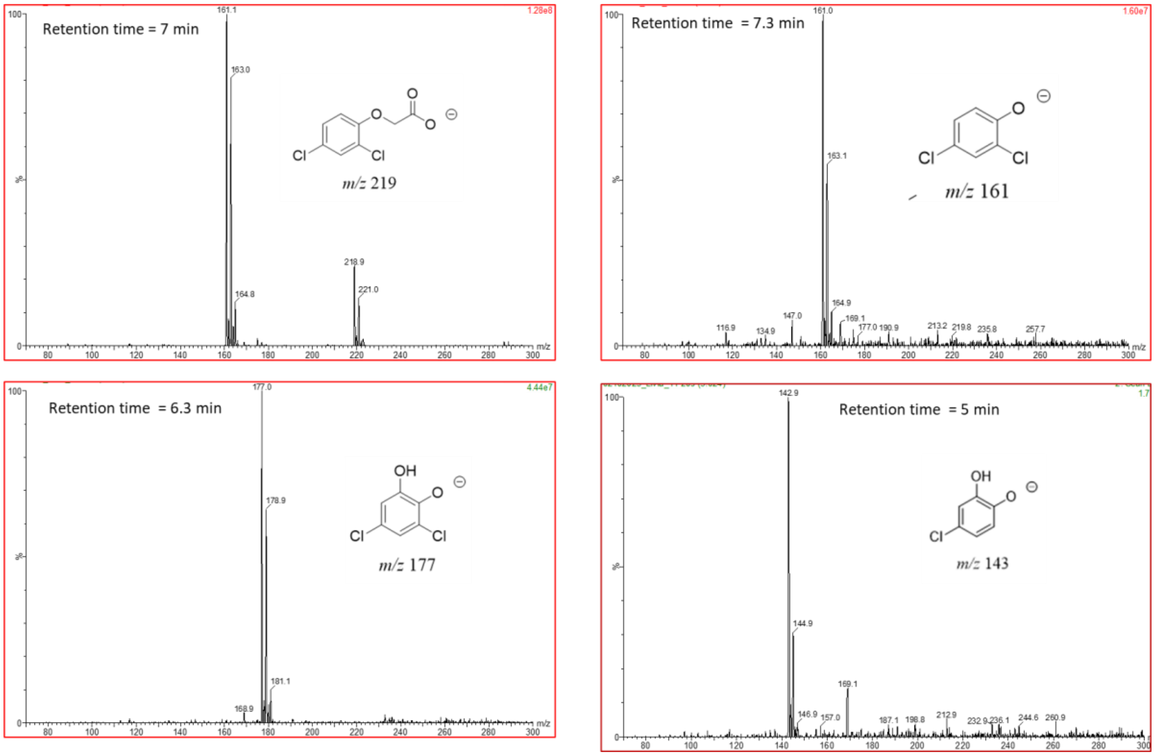

Fig. S9: Mass spectra of the three major chromatographic peaks (T_R= 7, 7.3, 6.3 and 5 min) in Fig. S7 along with corresponding structure of the compounds.

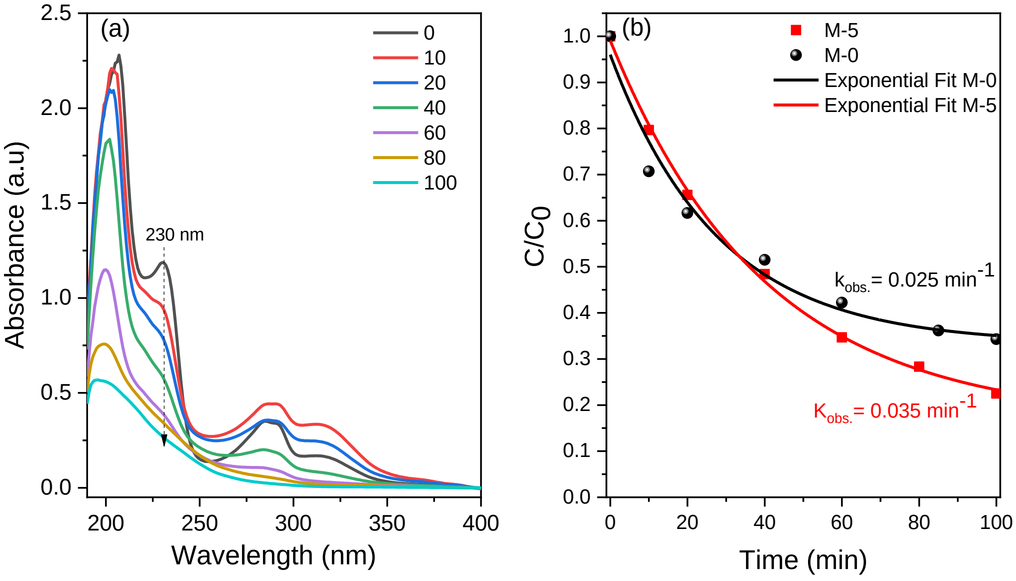

Fig. S10: (a) Electronic absorption spectra of 2,4-D and (b) kinetic profiles for the photodegradation of 2,4-D herbicide as function of illumination time in the presence of M-5 sample. Condition: 2,4-D initial concentration = 10 mg/L, photocatalyst amount = 1g/L, Light source= Xenon lamp

S6: PHYSIOCHEMICAL CHARACTERTISTIC OF M-ABC-5 SAMPLE PREPARED WITH NH_4HCO_3 AS GASEOUS TEMEPLATE

S6.1: *TEM Study*

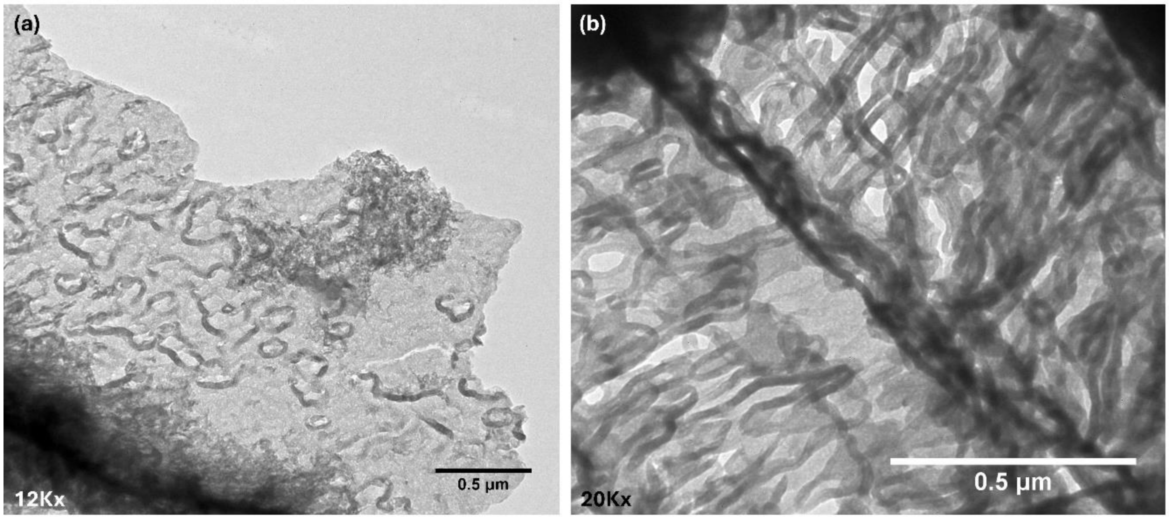

Fig. S11. TEM images of ABC-5-OE sample prepared with NH_4HCO_3 as dynamic gaseous template in different magnifications: (a) 12Kx (b) 20Kx

S6.2: *DRS Study*

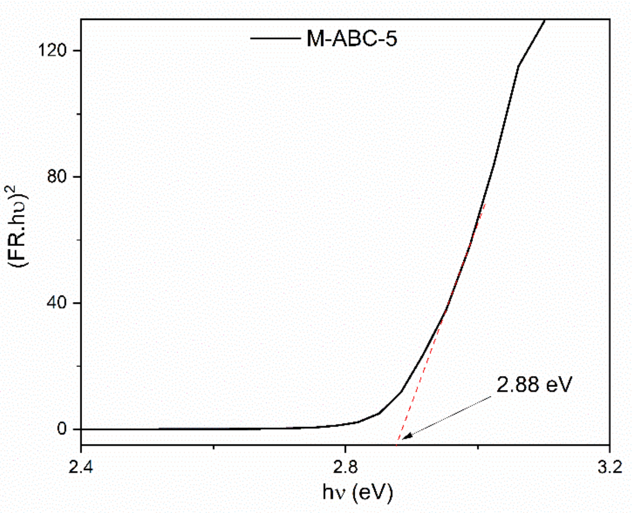

Fig. S12: Tauc's plot, showing the band gap (E_g) of g-C_3N_4 samples (M-ABC-5) prepared with NH_4HCO_3.

S6.3: *Lifetime Measurement (PL study)*

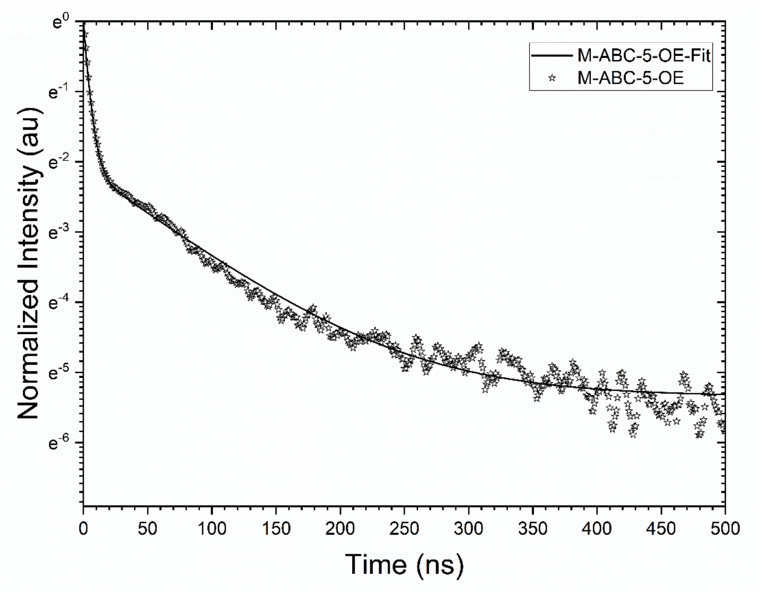

Fig. S13: Fitting of the time-resolved recombination fluorescence decay curves for M-ABC-5 sample. The g-C_3N_4 samples was photoexcited at wavelength of 375 nm from a picosecond pulsed LED source. The calculated average lifetime for this sample is 55 ns.

S6.4: *Photocatalytic Activity*

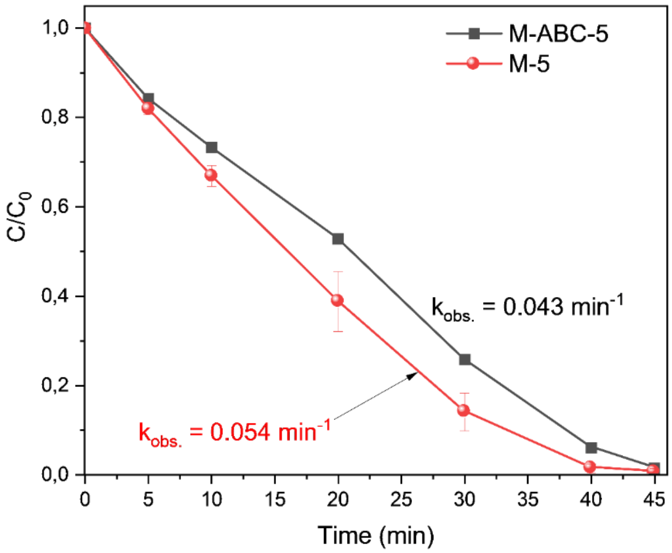

Fig. S14: A comparison of the kinetic profiles and observed rate constants ($k_{obs.}$) for M-5 and M-ABC-5 samples prepared with the same amounts (5 g each) of NH_4Cl and NH_4HCO_3, respectively

S7: Nitrogen Physisorption measurements

Adsorption and desorption of nitrogen gas isotherms of M-0, M-5 and M-10 before and after OE treatment are shown in Fig. S15.

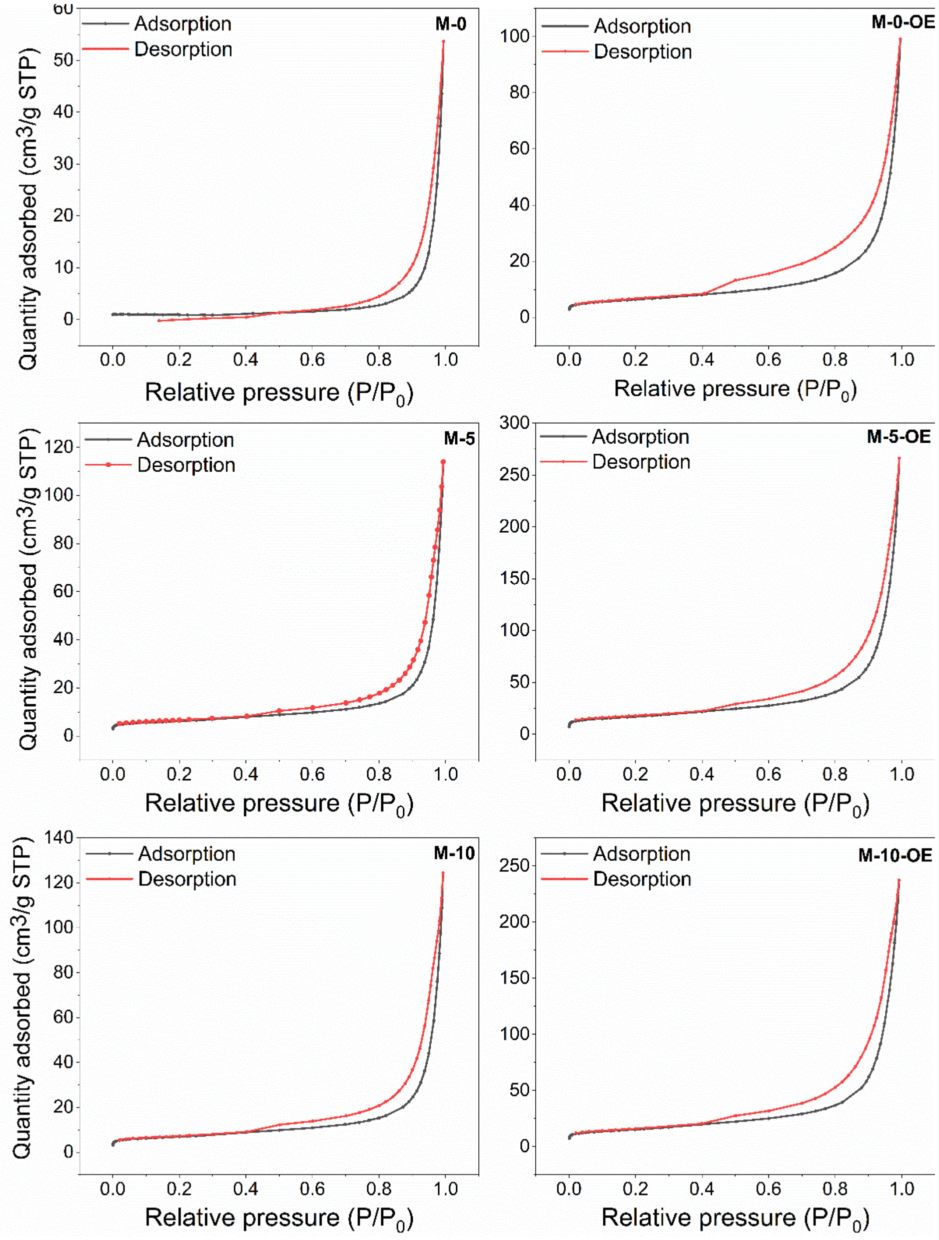

Fig. S15: Adsorption and desorption nitrogen gas isotherms obtained by N_2 physisorption experiments on M-0, M-5 and M-10 samples before (left column) and after (right column) OE treatment.

By comparing and analyzing the measured and standard isotherms and hysteresis loops according to IUPAC report (2015) [2], it is clear that the adsorption-desorption isotherms of

the samples are alike, which are of type IV, characterizing mesoporous materials [2]. From the isotherms, it can be observed that when the relative pressure $P/P_0 > 0.4$, the desorption and the absorption isotherms do not coincide, and the adsorption hysteresis loop appears, which is consistent with an IV(a) type curve. The type of hysteresis loop reflects the pore structure present in the adsorbent, and for all samples the types of hysteresis loop in low-temperature nitrogen adsorption experiments are a combination of H3 and H4 types. For the M-0 sample, an H3-type hysteresis loops is observed which is characteristic of slit-like pores that are often exhibited by aggregates of clays and lamellar materials. On the other hand, for the rest of the samples H4-type hysteresis loops occur, typical of micropores and mesopores materials and are often observed in micro-mesoporous carbons [2].

S8: Steady-state Photoluminescence (PL) study

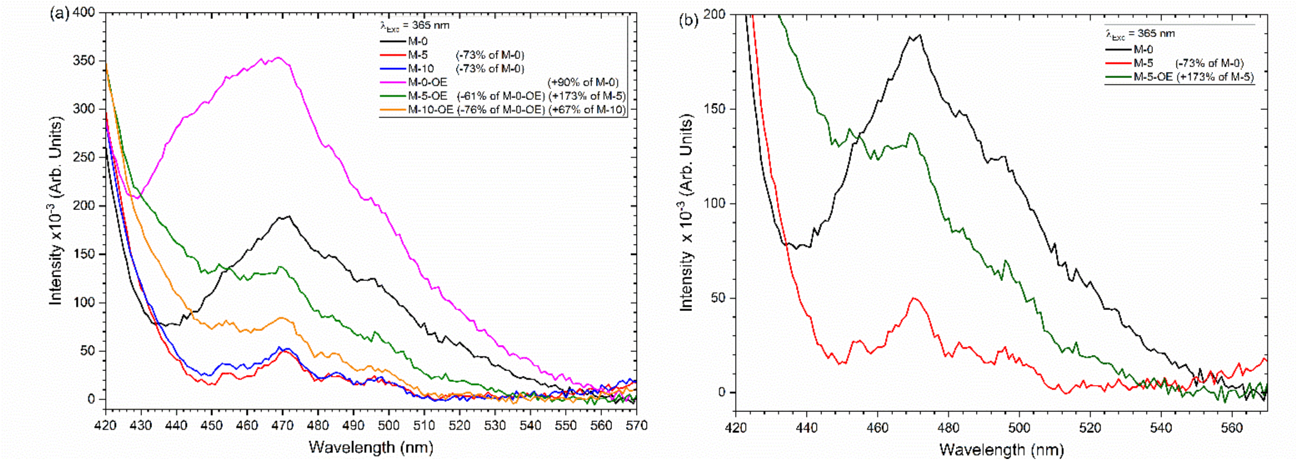

Figure S16. Steady-state PL emission spectra (under 365 nm excitation) of M-0 compared to (a) all EmNs samples before and after OE and (b) selected EmNs samples (M-5, and M-5-OE).

S9: EDS analysis of N content of EmNS

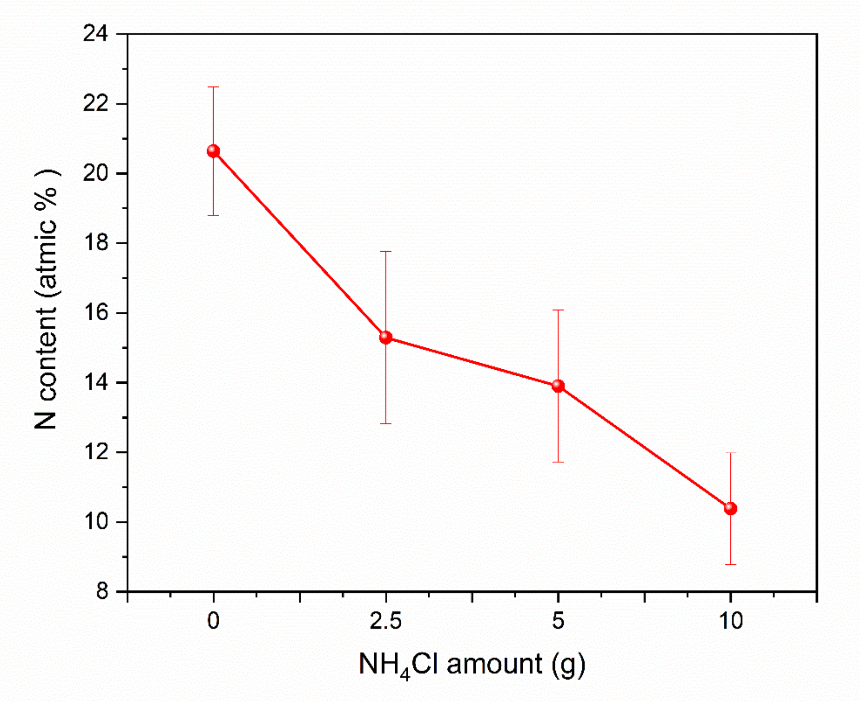

Fig. S17: EDS analysis showing the decrease in N content (atomic %) of the samples with increase in the amount (g) of NH_4Cl.

S10: EPR analysis of M-0 and EmNs samples before and after OE treatment

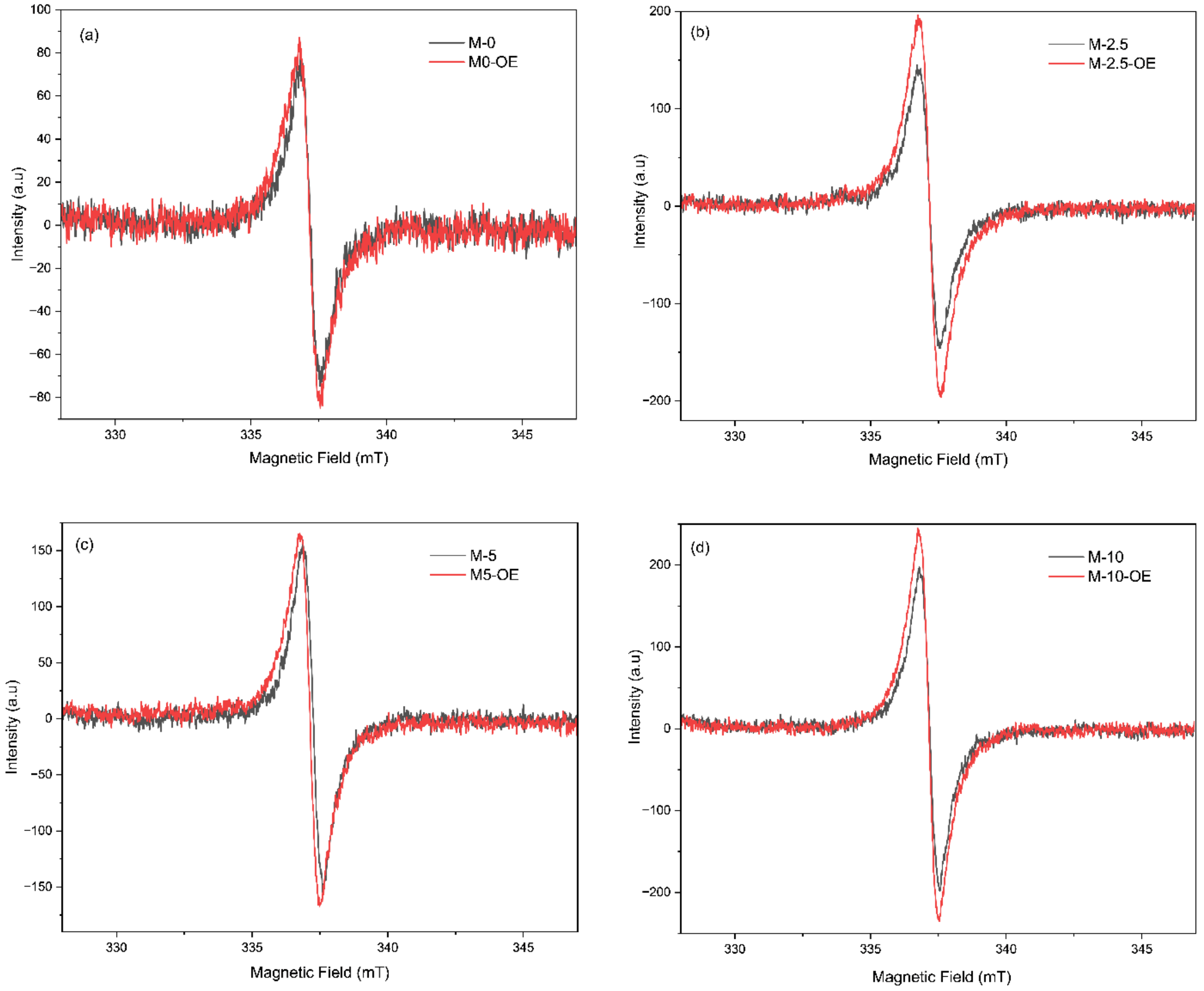

Fig. S18: EPR spectra of g-C_3N_4 samples before (black curves) and after (red curves) OE treatment indicating an increase in number of N-vacancies after OE treatment; (a) M-0 vs. M-0-OE, (b) M-2.5 vs. M-2.5-OE, (c) M5 vs. M5-OE (d) M-10 vs. M-10-OE.

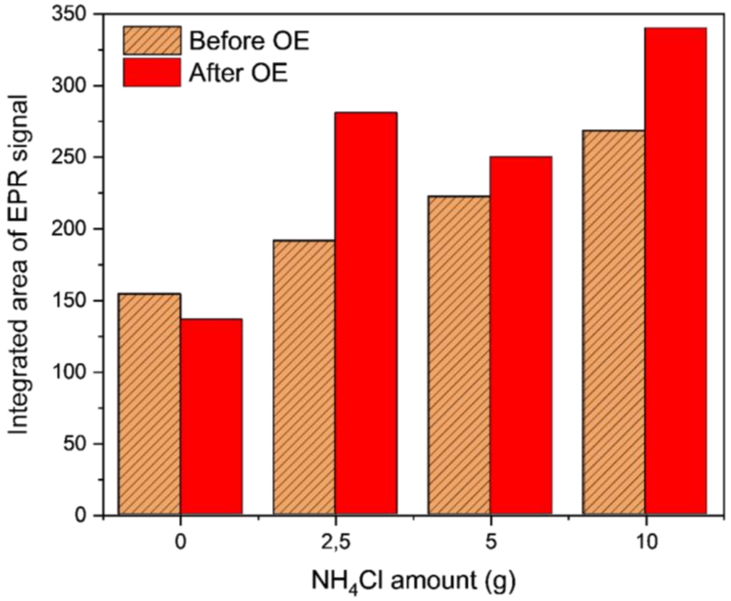

Fig. S19: Comparison of the integrated area of EPR signal of g-C_3N_4 samples as function of OE treatment. The integrated area before (dashed curves) and after (solid bars) OE treatment clearly indicates an increase in number of N-vacancies after OE treatment.

S11: XPS analysis of EmNS samples

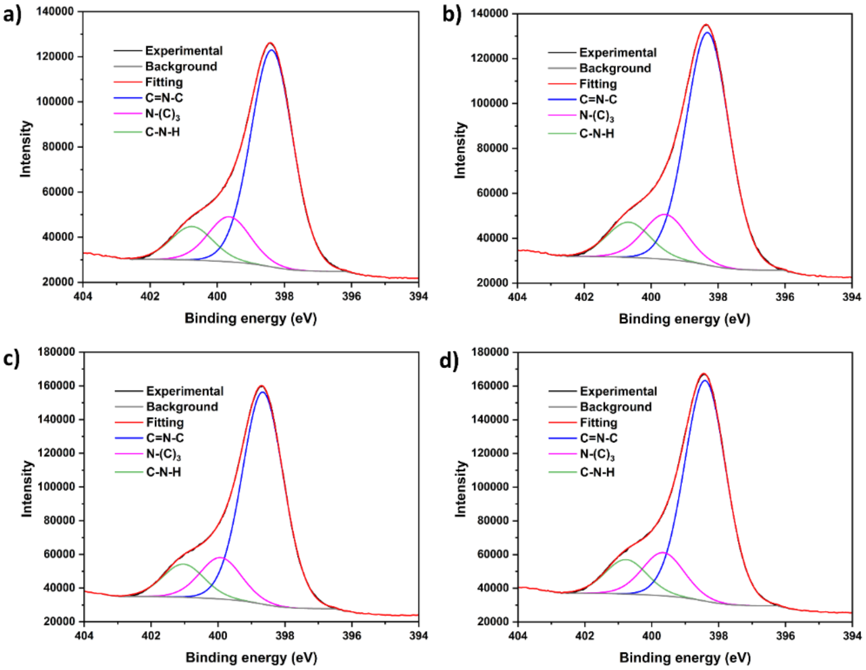

Fig. S20: High resolution XPS spectrum of the N1s core region; (a) M-0, (b) M-0-OE, (c) M5 and (d) M-5-OE samples, deconvoluted to show contributions of C=N-C, N-$(C)_3$ and C-N-H nitrogen chemical species of g-C_3N_4.

Table S1: Relative concentration of nitrogen surface chemical species of g-C_3N_4 obtained from deconvolution of N 1s XPS spectra

Sample	C–N=C	N–(C)$_3$	C-N-H
M-0	73.45 %	15.22 %	11.33 %
M-0-OE	74.45 %	14.32 %	11.23 %
M-5	74.04 %	14.43 %	11.53 %
M-5-OE	74.00 %	14.46%	11.53 %

S12: Recyclability and Stability Study of EmNs

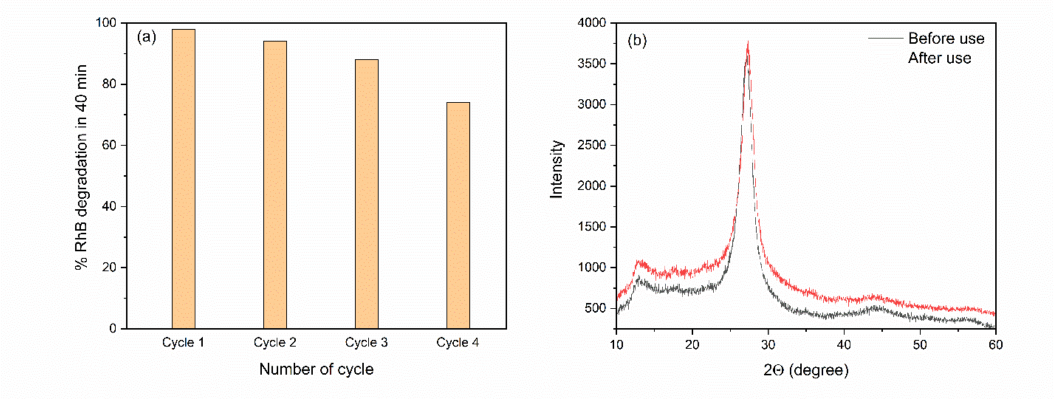

Fig. S21: Comparison of the % photodegradation of RhB after repeated photocatalytic cycles showing the recyclability of M-10 EmNs sample (a) and comparison of the XRD pattern of the photocatalyst before use and after 4 photocatalytic cycles.

S13: Synergistic effect for enhancing the physiochemical properties of EmNs: A summary

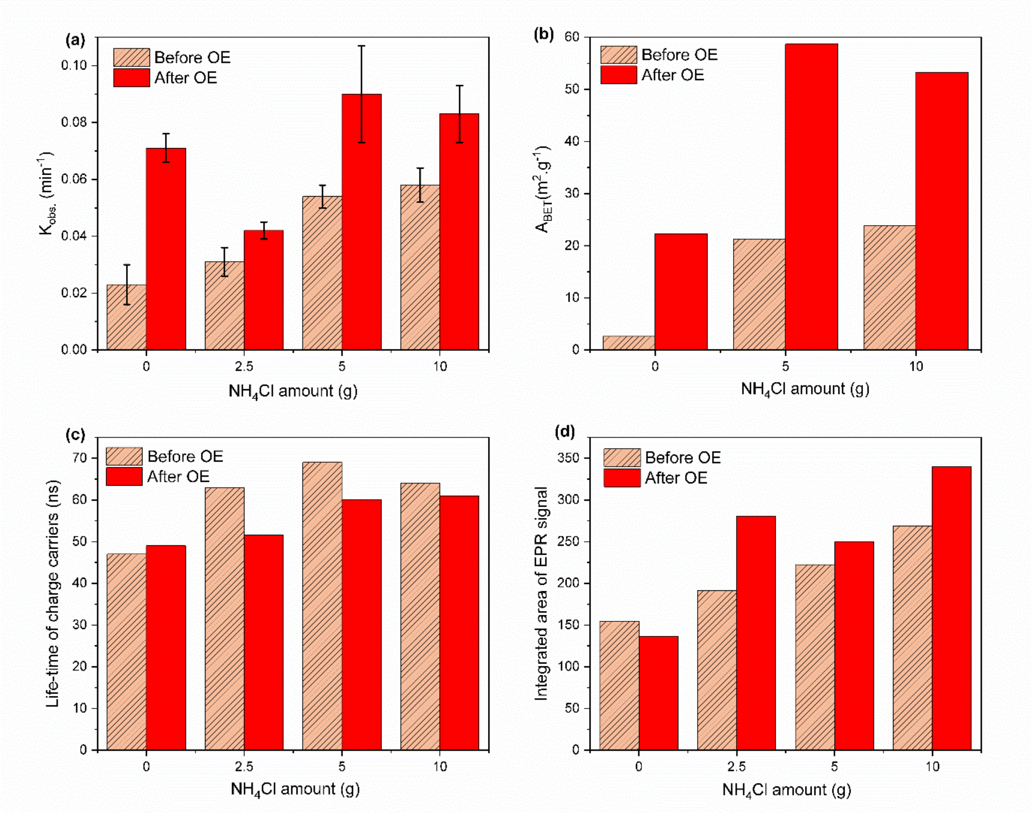

Fig. S22: Figure summarizing the synergistic effect of NH_4Cl and OE on the (a) photodegradation rate constant, $k_{obs.}$, (b), specific surface area, A_{BET} (c), lifetime of charge carriers and (d) density of nitrogen (N)-vacancies

S14: Mechanistic study using radical Scavengers

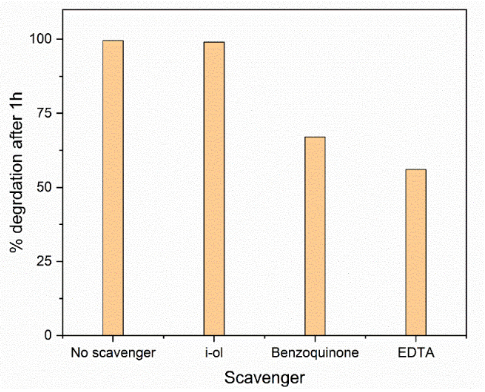

Fig. S23: Comparison of the % photodegradation of RhB in the absence and presence of different scavengers including isopropanol (i-ol), benzoquinone and EDTA as $^{\bullet}OH$ radicals, $O_2^{\bullet -}$ radical and h^+ scavengers, respectively.

Table S2: Comparison of the photoactivity of our EmNs samples with some other g-C_3N_4 based materials reported in the literature.

Photocatalyst	Light source	RhB (mg/L)	Photocatalyst (g/L)	degradation %	degradation time, min	Rate constant ($k_{obs.}$ min^{-1})	Ref.
g-C_3N_4	300 W Xe lamp, λ > 420 nm	10	0.2	84	60	0.029	[3]
g-C_3N_4 nanosheets	300 W Xe lam λ > 420 nm	10	1	100	30	0.15	[4]
g-C_3N_4 nanosheets	300 W Xe lamp λ > 420 nm	10	0.1	98	120	0.02	[5]
g-C_3N_4 nanosheets	400 W Xe Lamp	10	0.5	100	30	0.09	This study
g-C_3N_4 nanosheets	Sunlight	4.8	0.5	81 %	90 min	0.011	[6]
g-C_3N_4 nanosheets	Sunlight	10	0.5	100	18 min	0.16	This study
g-C_3N_4-$BiVO_4$	50 W LED λ > 360 nm	8.7	1.5	86	60 min	0.027	[7]
gC_3N_4/CNTs	300 W Xe lamp, λ > 420 nm	10	0.2	98	60	0.051	[3]
Ag@g-C_3N_4	150 W LED light	10	0.4	94	75	0.039	[8]

S15: References

1. Cao Y, Wu W, Wang S, et al (2016) Monolayer g-C3N4 Fluorescent Sensor for Sensitive and Selective Colorimetric Detection of Silver ion from Aqueous Samples. J Fluoresc 26:739–744. https://doi.org/10.1007/s10895-016-1764-9

2. Thommes M, Kaneko K, Neimark A V., et al (2015) Physisorption of gases, with special reference to the evaluation of surface area and pore size distribution (IUPAC Technical Report). Pure and Applied Chemistry 87:1051–1069. https://doi.org/10.1515/pac-2014-1117

3. Liu G, Liao M, Zhang Z, et al (2020) Enhanced photodegradation performance of Rhodamine B with g-C3N4 modified by carbon nanotubes. Sep Purif Technol 244:116618. https://doi.org/10.1016/j.seppur.2020.116618

4. Zhang R, Zhang X, Liu S, et al (2021) Enhanced photocatalytic activity and optical response mechanism of porous graphitic carbon nitride (g-C3N4) nanosheets. Mater Res Bull 140:111263. https://doi.org/10.1016/j.materresbull.2021.111263

5. Yan X, Kang B, Ai T, et al (2022) Enhanced visible light photocatalytic performance of crystalline g-C3N4 nanosheets by one-step molten salt method. Inorg Chem Commun 137:109209. https://doi.org/10.1016/j.inoche.2022.109209

6. Banu A, Sinha B, Sikdar S (2024) Synthesis of polymeric 2D-graphitic carbon nitride (g-C3N4) nanosheets for sustainable photodegradation of organic pollutants. Heliyon 10:e33354. https://doi.org/10.1016/j.heliyon.2024.e33354

7. Rohilla P, Pal B, Das RK (2023) Improved photocatalytic degradation of rhodamine B by g-C3N4 loaded BiVO4 nanocomposites. Heliyon 9:e21900. https://doi.org/10.1016/j.heliyon.2023.e21900

8. Luu Thi LA, Trieu QT, Trinh TH, et al (2025) Environmentally friendly fabrication of Ag nanoparticles decorated on g-C 3 N 4 for enhancing the photodegradation of RhB. Nanoscale Adv 7:5250–5261. https://doi.org/10.1039/D5NA00552C
